# Supplementary material for: Isoprene chemistry under upper-tropospheric conditions
Source: Nat Commun. 2025 Sep 29;16:8555. doi: 10.1038/s41467-025-64229-w (PMC12479858; doi:10.1038/s41467-025-64229-w)
Supplement: Supplementary file 1 — Supplementary information [file 41467_2025_64229_MOESM1_ESM.pdf]

Supplementary Information for "Isoprene chemistry under upper-tropospheric conditions"

Douglas M. Russell[1*], Felix Kunkler[2], Jiali Shen[3, 4], Matthias Kohl[2], Jenna DeVivo[5, 6], Nirvan Bhattacharyya[5, 6], Christos Xenofontos[7], Hannah Klebach[1], Lucía Caudillo-Plath[1], Mario Simon[1], Emelda Ahongshangbam[8, 3], Joao Almeida[9, 10], Antonio Amorim[10, 11], Hannah Beckmann[12], Mattia Busato[9], Manjula Canagaratna[13], Anouck Chassaing[14], Romulo Cruz-Simbron[15, 16], Lubna Dada[17], Philip Holzbeck[2], Bernhard Judmaier[18], Milin Kaniyodical Sebastian[19], Paap Koemets[12], Timm Krüger[1], Lu Liu[17], Monica Martinez[2], Bernhard Mentler[18], Aleksandra Morawiec[20], Antti Onnela[9], Tuukka Petäjä[3], Pedro Rato[9, 1], Mago Reza[15, 16], Samuel Ruhl[2], Wiebke Scholz[18], Eva Sommer[9, 20], António Tomé[21], Yandong Tong[15, 16], Jens Top[17], Nsikanabasi Silas Umo[19], Gabriela R. Unfer[22], Ryan X. Ward[23], Jakob Weissbacher[18], Boxing Yang[17], Wenjuan Yu[3], Marcel Zauner-Wieczorek[1], Imad Zgheib[24], Jiangyi Zhang[3], Zhensen Zheng[18, 25], Imad El Haddad[17], Richard C. Flagan[23], Armin Hansel[18], Heikki Junninen[12], Markku Kulmala[3, 4, 26, 27], Katrianne Lehtipalo[3, 28], Jos Lelieveld[2, 7], Ottmar Möhler[19], Siegfried Schobesberger[29], Rainer Volkamer[15, 16], Paul M. Winkler[20], Douglas R. Worsnop[13, 3], Theodoros Christoudias[7], Andrea Pozzer[2, 7], Neil M. Donahue[5, 6, 30], Hartwig Harder[2], Jasper Kirkby[1, 9], Xu-Cheng He[3, 31], and Joachim Curtius[1*]

[1] *Institute for Atmospheric and Environmental Sciences, Goethe University Frankfurt, 60438 Frankfurt am Main, Germany*
[2] *Atmospheric Chemistry Department, Max Planck Institute for Chemistry, Mainz, Germany*
[3] *Institute for Atmospheric and Earth System Research/Physics, Faculty of Science, University of Helsinki, Helsinki, Finland*
[4] *Helsinki Institute of Physics, University of Helsinki, Helsinki, Finland.*
[5] *Department of Chemistry, Carnegie Mellon University, 5000 Forbes Ave Pittsburgh, PA 15213 USA*
[6] *Center for Atmospheric Particle Studies, Carnegie Mellon University, Pittsburgh, PA 15213 USA*
[7] *Climate and Atmosphere Research Center (CARE-C), The Cyprus Institute, 1645 Nicosia, Cyprus*
[8] *Department of Chemistry, University of Helsinki, Helsinki 00014, Finland*
[9] *CERN, European Organisation for Nuclear Research, 1211 Geneva, Switzerland*
[10] *Faculdade de Ciências da Universidade de Lisboa, Campo grande, Ed. C8, Lisboa, Portugal*
[11] *Laboratório de Instrumentação e física experimental de Partículas, Portugal*
[12] *Institute of Physics, University of Tartu, W. Ostwaldi Str 1, Tartu, Estonia*
[13] *Aerodyne Research Inc., Billerica, MA, USA*
[14] *Department of Environmental Science, Stockholm University, Stockholm, Sweden*
[15] *Department of Chemistry, University of Colorado Boulder, Boulder, CO, USA*
[16] *Cooperative Institute for Research in Environmental Sciences, University of Colorado Boulder, Boulder, CO, USA*
[17] *Center for Energy and Environmental Sciences, Paul Scherrer Institute, Villigen, Switzerland*
[18] *Institute for Ion Physics and Applied Physics, University of Innsbruck, Innsbruck, Austria*
[19] *Institute of Meteorology and Climate Research, Atmospheric Aerosol Research, Karlsruhe Institute of Technology, Karlsruhe, Germany*
[20] *Faculty of Physics, University of Vienna, Wien, Austria*
[21] *IDL-Universidade da Beira interior, Rua Marquês D'ávila e Bolama 6201-001 Covilhã, Portugal*
[22] *Atmospheric Microphysics Department, Leibniz Institute for Tropospheric Research, Leipzig, Germany*
[23] *Division of Chemistry and Chemical Engineering, California Institute of Technology, Pasadena, CA, USA*
[24] *TOFWERK AG, Schorenstrasse 39, 3645, Thun, Switzerland*
[25] *IONICON Analytik GmbH, 6020 Innsbruck, Austria*
[26] *Joint International Research Laboratory of Atmospheric and Earth System Sciences, School of Atmospheric Sciences, Nanjing University, Nanjing, China.*
[27] *Aerosol and Haze Laboratory, Beijing Advanced Innovation Center for Soft Matter Science and Engineering, Beijing University of Chemical Technology, Beijing, China.*
[28] *Finnish Meteorological Institute, Helsinki, Finland*
[29] *Department of Technical Physics, University of Eastern Finland, PO Box 1627, 70211 Kuopio, Finland*
[30] *Department of Chemical Engineering, Carnegie Mellon University, Pittsburgh, PA 15213 USA*
[31] *Yusuf Hamied Department of Chemistry, University of Cambridge, Cambridge, UK.*
[*] *Corresponding authors: D. M. Russell (russell@iau.uni-frankfurt.de), J. Curtius (curtius@iau.uni-frankfurt.de)*

Supplementary Information

A Reaction Scheme

Wennberg et al. [1] describes a comprehensive compilation of gas-phase isoprene reactions, with detailed isomeric pathways. Given the instruments available, it is difficult to determine isomeric fine structure for IP-OOM, therefore the reaction scheme has been simplified to sum formula. The main reactions considered in this study are listed below and describe multiple isoprene $OH^{\bullet}$ oxidations coupled with $NO^{\bullet}$, $HO_2^{\bullet}$ and $NO_2^{\bullet}$ termination. Additional pathways to reach the sum formula of these compounds, including $NO_3^{\bullet}$ oxidation can occur. However, under the CLOUD conditions it has been shown that these were the dominant reactions.

$$C_5H_8 + OH^{\bullet} \xrightarrow{k_{OH}} C_5H_9O^{\bullet}$$

$$C_5H_9O^{\bullet} + O_2 \xrightarrow{k_{O_2}} ISOPOO^{\bullet}$$

$$ISOPOO^{\bullet} + HO_2^{\bullet} \xrightarrow{k_{HO_2}} ISOPOOH$$

$$ISOPOO^{\bullet} + NO^{\bullet} \xrightarrow{k_{NO}} IHN + MVK/MACR$$

$$ISOPOO^{\bullet} + NO_2^{\bullet} \xrightarrow{k_{NO_2}} IHPN$$

$$ISOPOOH + OH^{\bullet} \xrightarrow{k_{OH}} C_5H_{11}O_4^{\bullet}$$

$$ISOPOOH + OH^{\bullet} \xrightarrow{k_{OH}} IEPOX + OH^{\bullet}$$

$$IHN + OH^{\bullet} \xrightarrow{k_{OH}} C_5H_{10}NO_5^{\bullet}$$

$$IHN + OH^{\bullet} \xrightarrow{k_{OH}} IEPOX + NO_2^{\bullet}$$

$$IHPN + OH^{\bullet} \xrightarrow{k_{OH}} C_5H_{10}NO_6^{\bullet}$$

$$C_5H_{11}O_4^{\bullet} + O_2 \xrightarrow{k_{O_2}} C_5H_{11}O_6^{\bullet}$$

$$C_5H_{10}NO_5^{\bullet} + O_2 \xrightarrow{k_{O_2}} C_5H_{10}NO_7^{\bullet}$$

$$C_5H_{10}NO_6^{\bullet} + O_2 \xrightarrow{k_{O_2}} C_5H_{10}NO_8^{\bullet}$$

$$C_5H_{11}O_6^{\bullet} + HO_2^{\bullet} \xrightarrow{k_{HO_2}} C_5H_{12}O_6$$

$$C_5H_{11}O_6^{\bullet} + NO^{\bullet} \xrightarrow{k_{NO}} C_5H_{11}NO_7$$

$$C_5H_{11}O_6^{\bullet} + NO^{\bullet} \xrightarrow{k_{NO}} C_5H_{11}O_5^{\bullet} + NO_2^{\bullet}$$

$$C_5H_{11}O_6^{\bullet} + NO_2^{\bullet} \xrightarrow{k_{NO_2}} C_5H_{11}NO_8$$

$$C_5H_{10}NO_7^{\bullet} + HO_2^{\bullet} \xrightarrow{k_{HO_2}} C_5H_{11}NO_7$$

$$C_5H_{10}NO_7^{\bullet} + NO^{\bullet} \xrightarrow{k_{NO}} C_5H_{10}N_2O_8$$

$$C_5H_{10}NO_7^{\bullet} + NO^{\bullet} \xrightarrow{k_{NO}} C_5H_{10}NO_6^{\bullet} + NO_2^{\bullet}$$

$$C_5H_{10}NO_7^{\bullet} + NO_2^{\bullet} \xrightarrow{k_{NO_2}} C_5H_{10}N_2O_9$$

$$C_5H_{10}NO_8^{\bullet} + HO_2^{\bullet} \xrightarrow{k_{HO_2}} C_5H_{11}NO_8$$

$$C_5H_{10}NO_8^{\bullet} + NO^{\bullet} \xrightarrow{k_{NO}} C_5H_{10}N_2O_9$$

$$C_5H_{10}NO_8^{\bullet} + NO^{\bullet} \xrightarrow{k_{NO}} C_5H_{10}NO_7^{\bullet} + NO_2^{\bullet}$$

$$C_5H_{10}NO_8^{\bullet} + NO_2^{\bullet} \xrightarrow{k_{NO_2}} C_5H_{10}N_2O_{10}$$

On the note of isomers, it is important to consider the intramolecular rearrangement between isomeric ISOPOOH and IEPOX

when considering the $C_5H_{10}O_3$ signal, as they are indistinguishable in a mass spectrometer and have quite important and different oxidation pathways. In Shen and Russell et al. 2024, a fit (Equation S1) was established between IEPOX fraction and $OH^•$ concentration, using model calculations. In this work, the ISOPOOH and IEPOX concentrations have been calculated using this relationship:

$$f_{\mathrm{IEPOX}} = 10^{0.56 \cdot \log_{10}[\mathrm{OH}^\bullet]-4.4} \tag{S1}$$

With the f_{ISOPOOH} fraction being 1- f_{IEPOX}.

Importantly, detection of $C_5H_{10}O_3$ in the Br-MION2-CIMS is similar to HOI rather than H_2SO_4, therefore, a calibration factor two times that of H_2SO_4 was used for this species[2]. However, all the IP-OOM from Br-MION2-CIMS and NO_3-CIMS use a calibration for H_2SO_4 as laid out in the Methods.

In addition to chemical reactions, there are processes inherent to CLOUD that need to be considered, such as the loss rate (k_L) a combination of loss to the chamber walls (k_{wall}) and the condensation sink to pre-existing aerosol. Species with $n_{\mathrm{O}} \geq 3$ will have $k_{\mathrm{wall}} = 1.6 \times 10^{-3}$ s^{-1} equivalent to that of sulfuric acid (SA) and assuming a perfect sink at 223 K [3].

B Peroxynitrate kinetics

The reaction of peroxy radicals with $NO_2^\bullet$ to form peroxynitrates (RO_2NO_2) is often neglected in atmospheric mechanisms as most peroxynitrates are thought to be thermally unstable and consequently assumed to rapidly decay back to their radical precursors. The well known exceptions are peroxy acetylnitrates (PANs) which have been well studied and are atmospherically important species [4]. Specifically, PANs become progressively more stable as temperatures drop and thus are able to carry NO_x from warm, NO_x rich conditions of the planetary boundary layer into the remote troposphere. However, outside of PAN formation, there is very little kinetic information about the reaction of $RO_2^\bullet + NO_2^\bullet \longrightarrow RO_2NO_2$, due to the rapid thermal decomposition of $RO_2NO_2 \longrightarrow RO_2^\bullet + NO_2^\bullet$. At temperatures (223 & 243 K) achievable in the CLOUD chamber, RO_2NO_2 are stable long enough to measure; as a result, we can determine important information regarding their kinetics. Further, at these same temperatures in the upper troposphere, their lifetimes may be long enough so decomposition is not the only important sink – for example, condensation to particle may become important. Assuming that thermal decomposition is negligible at these cold temperatures, the steady-state kinetic equations for $C_5H_{10}N_2O_{10}$, $C_5H_{10}N_2O_9$ and $C_5H_{10}N_2O_8$ can be expressed as:

$$\frac{d[C_5H_{10}N_2O_{10}]}{dt} = k_{NO_2}[NO_2^\bullet][C_5H_{10}NO_8^\bullet]-k_L[C_5H_{10}N_2O_{10}] \tag{S2}$$

$$\frac{d[C_5H_{10}N_2O_9]}{dt} = k_{NO_2}[NO_2^\bullet][C_5H_{10}NO_7^\bullet] + \alpha_{12}k_{NO}[NO^\bullet][C_5H_{10}NO_8^\bullet]-k_L[C_5H_{10}N_2O_9] \tag{S3}$$

$$\frac{d[C_5H_{10}N_2O_8]}{dt} = \alpha_{11}k_{NO}[NO^\bullet][C_5H_{10}NO_7^\bullet]-k_L[C_5H_{10}N_2O_8] \tag{S4}$$

α_i denotes an organonitrate branching ratio of an $RO_2^\bullet$ with i non-hydrogen atoms in the R-group. We can solve Equation S2, Equation S3 and Equation S4 for $[C_5H_{10}NO_8^\bullet]$ and $[C_5H_{10}NO_7^\bullet]$ under steady-state conditions to give Equation S5 and Equation S6.

$$[C_5H_{10}NO_8^\bullet]_{SS} = \frac{k_L[C_5H_{10}N_2O_{10}]}{k_{NO_2}[NO_2^\bullet]} = \frac{k_L[C_5H_{10}N_2O_9]-k_{NO_2}[NO_2^\bullet][C_5H_{10}NO_7^\bullet]}{\alpha_{12}k_{NO}[NO^\bullet]} \tag{S5}$$

$$[C_5H_{10}NO_7^\bullet]_{SS} = \frac{k_L[C_5H_{10}N_2O_8]}{\alpha_{11}k_{NO}[NO^\bullet]} \tag{S6}$$

Lifetimes of $RO_2^\bullet$ in the chamber are short in comparison to closed-shell molecules in the presence of high NO_x (see Figure S2), and $RO_2^\bullet$ concentrations of the chamber may be underestimated compared to closed-shell molecules. Therefore, we can substitute Equation S5 into Equation S6 and simplify to give a quadratic equation, Equation S7, with variables that we can measure confidently.

$$\beta^2\frac{[C_5H_{10}N_2O_8]}{\alpha_{11}\alpha_{12}}-\beta\frac{[C_5H_{10}N_2O_9]}{\alpha_{12}} + [C_5H_{10}N_2O_{10}] = 0 \tag{S7}$$

Where $\beta = \frac{k_{NO_2}[NO_2^\bullet]}{k_{NO}[NO^\bullet]}$. For all the steady-state stages, we have solved Equation S7 using the quadratic formula, to yield an array of β values. Figure S3 plots calculated β values against measured $\frac{[NO_2^\bullet]}{k_{NO}[NO^\bullet]}$ which would give a gradient equivalent to k_{NO_2}. There are two distinct lines due to the two roots of each solution to the quadratic equation. A quadratic equation arises as the sum formula we are considering are a combination of at least two isomers. Two distinct roots could indicate a variation in k_{NO_2} depending on

$RO_2^{\bullet}$, as we are assuming the rate coefficient for $C_5H_{10}NO_7^{\bullet}$ and $C_5H_{10}NO_8^{\bullet}$ + $NO_2^{\bullet}$ to be the same. In addition, this may reflect the variation in the α_i for each $RO_2^{\bullet}$ as it is well understood that this changes with i, further study would be required to determine the source of these two roots. The two fits are $\beta = 3.42 \times 10^{-12} \frac{[NO_2^{\bullet}]}{k_{NO}[NO^{\bullet}]} + 0.28$ and $\beta = 1.42 \times 10^{-12} \frac{[NO_2^{\bullet}]}{k_{NO}[NO^{\bullet}]} + 0.28$. Assuming the rate coefficient of k_{NO} to be accurate, we can estimate a value of k_{NO_2} = 3.42 × 10^{-12} cm^3s^{-1} and 1.42 × 10^{-12} cm^3s^{-1} for each fit, respectively. Using k_{NO_2} = 3.42 × 10^{-12} cm^3s^{-1} yields a $\frac{k_{NO}}{k_{NO_2}} \sim 3.8$, which agrees with the point of equal termination from Figure 4 (a). Literature values for the reaction rate coefficients of fluorinated C_{2-3} peroxy radicals with $NO^{\bullet}$ and $NO_2^{\bullet}$ as shown in Table S1 indicate the range of $\frac{k_{NO_2}}{k_{NO}}$ lies between 0.09 – 0.48, agreeing with our results that yield a value of 0.26. Tyndall et al. [5] suggests that for smaller peroxy radicals this ratio can even approach 1.

Performing similar analysis to calculate k_{HO_2}, using $C_5H_{12}O_6$, $C_5H_{11}NO_7$ and $C_5H_{10}N_2O_8$, yields 7.5 × 10^{-11} cm^3s^{-1}, which agrees with the rate coefficient from Wennberg et al. [1] of 7.2 × 10^{-11} cm^3s^{-1} at 223 K. Therefore, this method can be used to determine an estimate for the reaction rate coefficient of $RO_2^{\bullet}$ + $NO_2^{\bullet}$, in lieu of kinetic flow tube measurements.

C Reacted ISOPOOH, IEPOX, IHN, IHPN

Figure 2 (b) presents the yield of IP-OOM, IP_{0N}, IP_{1N} and IP_{2N} from "Reacted IP-OOM precursors". IP-OOM are defined as compounds with n_C,$n_O^{eff} > 3$, where $n_O^{eff} = n_O - 2 \cdot n_N$. IP_{0N}, IP_{1N} and IP_{2N} are subsets of IP-OOM, each containing differing amounts of nitrogen atoms. IP_{0N} are IP-OOM with functional groups that do not contain a N atom (n_N = 0, $n_C > 3$, $n_O > 3$), primarily containing –OH, –OOH and –O– functionality. IP_{1N} contain functional groups with exactly one N atom (n_N = 1, $n_C > 3$, $n_O > 5$), this will include $-ONO_2$ and $-O_2NO_2$ groups. IP_{2N} contain two functional groups which contain an N atom, (n_N = 2, $n_C > 3$, $n_O > 7$).

As laid out in the reaction scheme, IP-OOM are generally second generation $OH^{\bullet}$ oxidation products. Consequently, a dominant proportion of IP-OOM are oxidation products of the first-generation intermediates: ISOPOOH, IHN and IHPN. However, for the sake of completeness, IEPOX, an isomer of ISOPOOH which can also be oxidised further, will be included in this discussion as well. Therefore, IP-OOM precursors describes species which can be oxidised to form IP-OOM: ISOPOOH, IEPOX, IHN and IHPN.

Under steady-state conditions, the production and loss terms of IP-OOM and subsequently IP_{0N}, IP_{1N} and IP_{2N} are balanced. The overwhelming sink for IP-OOM in the CLOUD chamber are loss to the walls, where the wall loss rate has been measured as k_{wall} = 1.6 × $10^{-3}s^{-1}$ for sulfuric acid [6] which can be assumed to apply for all IP-OOM at this temperature [3]. The yield of IP-OOM can then be calculated from the reacted oxidation of IP-OOM precursors: ISOPOOH, IEPOX, IHN and IHPN with $OH^{\bullet}$. This yields the equation below:

$$[\text{IP-OOM}] \times k_{\text{wall}} = \text{Yield} \times \sum (k_X[X][OH^{\bullet}]) \\ X \in [\text{ISOPOOH, IEPOX, IHN, IHPN}] \tag{S8}$$

Reaction rate coefficients can be found in Table S2.

For each individual subset, we have additional information regarding their identity (n_N); therefore, we can create a more accurate calculation of their yields. For example, IP_{2N} can only be formed through IP-OOM precursors which contain a nitrogen atom, because in $OH^{\bullet}$ dominated addition, there is only the ability to add one nitrogen atom per round of oxidation. Therefore, precursors for IP_{2N} must be only IHN and IHPN. In contrast, to form IP_{0N}, the IP-OOM precursor cannot contain a nitrogen atom - under the kinetic chemical system we are observing it is highly unlikely that a nitrogen-containing functional group will be removed through an additional round of oxidation. If temperatures were higher, it would be possible for RO_2NO_2 to decompose and then this could happen; however, this is unlikely at 223 K. As a result, precursors for IP_{0N} are constrained to ISOPOOH and IEPOX. All precursors can contribute to IP_{1N} formation similar to IP-OOM formation. Consequently, we can modify Equation S8 for each subcase:

$$[IP_{0N}] \times k_{\text{wall}} = \text{Yield} \times (k_{\text{ISOPOOH}}[\text{ISOPOOH}][\text{OH}] + k_{\text{IEPOX}}[\text{IEPOX}][\text{OH}]) \tag{S9}$$

$$[IP_{1N}] \times k_{\text{wall}} = \text{Yield} \times (k_{\text{ISOPOOH}}[\text{ISOPOOH}][\text{OH}] + k_{\text{IEPOX}}[\text{IEPOX}][\text{OH}] \\ + k_{\text{IHN}}[\text{IHN}][\text{OH}] + k_{\text{IHPN}}[\text{IHPN}][\text{OH}]) \tag{S10}$$

$$[IP_{2N}] \times k_{\text{wall}} = \text{Yield} \times (k_{\text{IHN}}[\text{IHN}][\text{OH}] + k_{\text{IHPN}}[\text{IHPN}][\text{OH}]) \tag{S11}$$

However, using a different definition of the IP-OOM precursors for each subset does result in the sum of the subset yields differing from the yield of IP-OOM. Rearranging Equation S8, Equation S9, Equation S10 and Equation S11, we can form relationships between concentration of IP-OOM, IP_{0N}, IP_{1N} and IP_{2N} and their respective reacted IP-OOM precursor (RIP). Using reacted IP-OOM precursors gives an estimation of the expected concentration of IP-OOM, IP_{0N}, IP_{1N} and IP_{2N}. Comparing our measured

values to those expected in Figure 2 (b), we can determine the yield of IP-OOM and its subsets as a function of precursor oxidation, allowing for a simplification of the chemistry to be used in global models.

$$\mathrm{RIP}_{[\text{IP-OOM}]} = \frac{(k_{\mathrm{ISOPOOH}}[\mathrm{ISOPOOH}] + k_{\mathrm{IEPOX}}[\mathrm{IEPOX}] + k_{\mathrm{IHN}}[\mathrm{IHN}] + k_{\mathrm{IHPN}}[\mathrm{IHPN}])[\mathrm{OH}]}{k_{\mathrm{wall}}}$$

$$\mathrm{RIP}_{[\text{IP-OOM (No NO}_x)]} = \frac{(k_{\mathrm{ISOPOOH}}[\mathrm{ISOPOOH}] + k_{\mathrm{IEPOX}}[\mathrm{IEPOX}])[\mathrm{OH}]}{k_{\mathrm{wall}}}$$

$$\mathrm{RIP}_{[\mathrm{IP_{0N}}]} = \frac{(k_{\mathrm{ISOPOOH}}[\mathrm{ISOPOOH}] + k_{\mathrm{IEPOX}}[\mathrm{IEPOX}])[\mathrm{OH}]}{k_{\mathrm{wall}}}$$

$$\mathrm{RIP}_{[\mathrm{IP_{1N}}]} = \frac{(k_{\mathrm{ISOPOOH}}[\mathrm{ISOPOOH}] + k_{\mathrm{IEPOX}}[\mathrm{IEPOX}] + k_{\mathrm{IHN}}[\mathrm{IHN}] + k_{\mathrm{IHPN}}[\mathrm{IHPN}])[\mathrm{OH}]}{k_{\mathrm{wall}}}$$

$$\mathrm{RIP}_{[\mathrm{IP_{2N}}]} = \frac{(k_{\mathrm{IHN}}[\mathrm{IHN}] + k_{\mathrm{IHPN}}[\mathrm{IHPN}])[\mathrm{OH}]}{k_{\mathrm{wall}}}$$

This definition of IP-OOM was used to encapsulate isoprene oxidation products that have low enough volatility to contribute to growth of small aerosol at 223 K; there are no conditions enforcing compounds to have gone through two $OH^{\bullet}$ oxidations. Therefore, under low oxidation conditions, more IP-OOM may be formed through single oxidation than double oxidation ultimately resulting in an IP-OOM (or subset) yield greater than 100%.

D Composition of IP-OOM in case study

$RO_2^{\bullet}$ termination with different radicals can lead to drastically different compositions of IP-OOM. In three cases (HO_2, NO, and NO_2) we can discern the effects of different radical ratios. Grouping each case based on carbon backbone can yield some useful insights (Figure S4). When considering total organic signal, similar total concentrations are observed, with a $\pm 33\%$ spread between the cases, therefore, confirming that the level of oxidation are comparable as defined in the case study, despite the HO_2 case containing greater isoprene concentrations. Figure S4 b shows that this organic signal is split between fragments ($n_C < 5$) and monomers ($n_C = 5$), whereas the total fraction of dimers ($n_C > 5$) to total organics is small for all three cases; an increasing fraction of fragments for the NO_x stages is indicative of the fragmentation sequence, $RO_2^{\bullet} + NO^{\bullet} \longrightarrow RO^{\bullet} \longrightarrow C_4$. Conversely, considering the additional constraints of IP-OOM that all molecules must be $n_C > 3$ and $n_O^{eff} > 3$, there is a larger discrepancy in total IP-OOM concentration between cases, with a $\pm$ 61% spread between cases, mainly driven by injected isoprene levels differing by a factor of two for the HO_2 case, despite similar levels of $OH^{\bullet}$. However, the percentage of IP-OOM to total organics is 5.6%, 7.2% and 8.3% for NO_2, NO and HO_2 cases, respectively, indicating that the level of secondary oxidation are comparable between the cases.

Comparing total organic signal to IP-OOM, the fragment fraction decreases significantly in all cases, evidence that the majority of fragments are present in the form of MVK and MACR and their less oxygenated products, as expected. In addition, the low temperatures lead to greater $RO_2^{\bullet}$ stability and a higher preference for bimolecular reactions rather than fragmentation. The largest reduction in the fragment proportion was observed in the NO case, from 61 % to 4 %, which could also be an indication that multiple fragmentation reactions could be pushing the carbon content below the threshold ($n_C < 4$ or $n_O < 4$) towards more volatile species. Moreover, the NO_2 case retains an increased fragment fraction in IP-OOM; an intermediate $NO^{\bullet}$ concentration could cause some fragmentation but not enough for multiple fragmentation reactions to occur, therefore retaining a carbon backbone with n_C and n_O to be considered an IP-OOM. Using the same definition as Figure 2 (b), the yield of IP-OOM from reacted IP-OOM precursors are 32, 114 and 48 % for the HO_2, NO and NO_2 cases, respectively. Indicating that two $OH^{\bullet}$ oxidations may be more important for IP-OOM formation in the presence of $NO^{\bullet}$ than without.

A detailed breakdown of IP-OOM in Figure S5, displays the main species comprising the fragment, monomer, and dimer fractions of IP-OOM for each case. $C_4H_8O_4$, an oxidation product of MACR ($C_4H_6O + OH\ (O_2) \longrightarrow C_4H_7O_4 + HO_2 \longrightarrow C_4H_8O_4$), makes up the majority of C_4 in the HO_2 case with analogous compounds for $NO^{\bullet}$ and $NO_2^{\bullet}$ termination, $C_4H_7NO_5$ ($RONO_2$) and $C_4H_7NO_6$ (RO_2NO_2), respectively present in the NO_x cases. Similar to the C_5 compounds, the NO case yields a more diverse composition, highlighting its effect on the underlying $RO_2^{\bullet}$ composition.

In the C_5 fraction, a greater number of species make up more than 3.5 % of the signal, owing to the increased complexity of bimolecular $RO_2^{\bullet}$ chemistry. Species discussed in Figure 1 make up a large proportion, hence the focus of this study. Other compounds in the HO_2 case arise from $RO_2^{\bullet} + RO_2^{\bullet}$ and isomerisation reactions, such as $C_5H_{12}O_5$, $C_5H_{10}O_5$, and $C_5H_{10}O_6$. The NO case exhibits similar behaviour with the analogous respective counterparts $C_5H_{11}NO_6$, $C_5H_9NO_6$, and $C_5H_9NO_7$. Importantly, the IP_{0N} distribution is similar between HO_2 and NO_2 cases but drastically different in the NO case, hinting at the effect $RO_2^{\bullet} + NO^{\bullet} \longrightarrow RO^{\bullet} + NO_2^{\bullet}$ has on RO_x complexity.

This is also observable in the “dimer” fraction, which accounts for 5-15% of IP-OOM. Compositions shift dramatically from C_{10} dimers in the HO_2 case to C_{6-7} dimers in the NO_x cases. This dramatic shift in composition can help explain the reduced nucleation efficiency of the isoprene + NO_x system [3], as the contribution of C_{10} accretion products (ULVOC/ELVOC) to nucleation is high. A lower percentage of C_{10} in the NO_x cases could be reasoned with increased competition of C_5 $RO_2^\bullet$ and D’Ambro et al. [7] have also found C_{6-7} species dominating the “dimer” fragment. This could be due to C_{1-2} $RO_2^\bullet$ formed from fragmentation of $RO^\bullet$ can dimerise to form C_{6-7} association products or a combination of C_{3-4} $RO_2^\bullet$; however, due to the large contribution to nucleation, more work is warranted to test this hypothesis and to quantify the effect of $NO^\bullet$ on the underlying RO_x composition. Further study to describe the changing $RO_2^\bullet$ distribution and determine the $RO_2^\bullet$ + $R'O_2^\bullet$ reactions is also required.

This case study allows for the characterisation of nitrate and peroxynitrate contributions without chemical separation. We were able to identify four peroxynitrate species ($C_5H_9NO_5$, $C_5H_{10}N_2O_{10}$, $C_5H_{11}NO_8$ and $C_4H_7NO_6$) with high concentration. Further work would be required to identify more.

E Br-MION2-CIMS vs NO_3-CIMS

In general, the algorithm described in the Methods was used to determine concentrations of species contributing to IP-OOM, however, for Figure 4 the NO_3-CIMS measurement was used for all species despite the Br-MION2-CIMS exhibiting a higher sensitivity for some species. This decision was made as some of the highly oxidised dinitrogen containing compounds were not detected in the Br-MION2-CIMS and it was important for the sensitivity and performance of the instrument to be consistent throughout all of the experiments, therefore, using only one mass spectrometer seemed the best possible course of action.

Comparing the second-generation products for both instruments in Figure S6, indicates a nice agreement that lies close to the 1:1 line, therefore such a substitution is valid for Figure 4. In addition, a comparison of methane sulfonic acid (MSA), Figure S6 (f), indicates 1:1 sensitivity for the inorganic acid confirming the agreement between the two instruments and validating the H_2SO_4 calibration factor for each instrument.

F First-generation product distribution

First-generation products, IHN and ISOPOOH are the intermediary species formed from one $OH^\bullet$ oxidation and termination with $NO^\bullet$ and $HO_2^\bullet$, respectively. IHN and ISOPOOH share a common parent $RO_2^\bullet$, $ISOPOO^\bullet$, therefore, similar to Figure 4, the behaviour and ratio between these two compounds for a given $RO_2^\bullet$ concentration should be solely dependent on the $HO_2^\bullet$:$NO^\bullet$. Figure S7 presents this graphically, in a $HO_2^\bullet$ dominated environment ISOPOOH accounts for all the signal. Whereas in an $NO^\bullet$ dominated environment, IHN accounts $\sim 80\%$ of the normalised ISOPOOH and IHN distribution. Interestingly, ISOPOOH retains 20% of the signal even at the very low $HO_2^\bullet$:$NO^\bullet$, which could be evident of the inherent experimental connection between $OH^\bullet$ oxidation and $HO_2^\bullet$ termination - it is experimentally easier to achieve conditions without $NO^\bullet$. Moving from $NO^\bullet$ dominated to $HO_2^\bullet$ dominated regimes occurs rather suddenly, underlining the large changes small variations in radical ratios can have on the isoprene oxidation distribution within the transition regime. This occurs at a critical threshold of $HO_2^\bullet$:$NO^\bullet$ = 0.56. Comparing the termination radical ratio of second-generation species in Figure 4, 0.41, to a critical threshold of 0.56 yields a relatively good agreement. A larger critical threshold of 0.56 could be an indication that the second-generation $HO_2^\bullet$ to $NO^\bullet$ pathway may be relatively faster than the first-generation $HO_2^\bullet$ to $NO^\bullet$ pathway as a lower $HO_2^\bullet$:$NO^\bullet$ is required to achieve equal termination.

Unsaturated compounds, such as IHN and ISOPOOH, can undergo another oxidation step to form more highly oxidised compounds. Their steady-state rate equations can be expressed as Equation S12 and Equation S13.

$$\frac{d[\text{ISOPOOH}]}{dt} = k_{RO_2\ +\ HO_2}[HO_2^\bullet][\text{ISOPOO}^\bullet]\text{-}k_{OH\ +\ ISOPOOH}[OH^\bullet][\text{ISOPOOH}]\text{-}k_L[\text{ISOPOOH}] = 0 \tag{S12}$$

$$\frac{d[\text{IHN}]}{dt} = \alpha_6 k_{RO_2\ +\ NO}[NO^\bullet][\text{ISOPOO}^\bullet]\text{-}k_{OH\ +\ IHN}[OH^\bullet][\text{IHN}]\text{-}k_L[\text{IHN}] = 0 \tag{S13}$$

Using these two equations, we can determine two separate steady-state expressions for $[\text{ISOPOO}^\bullet]$, Equation S14:

$$[\text{ISOPOO}^\bullet]_{SS} = \frac{k_{OH\ +\ ISOPOOH}[OH^\bullet][\text{ISOPOOH}]\text{-}k_L[\text{ISOPOOH}]}{k_{RO_2\ +\ HO_2}[HO_2]} = \frac{k_{OH\ +\ IHN}[OH^\bullet][\text{IHN}]\text{-}k_L[\text{IHN}]}{\alpha_6 k_{RO_2\ +\ NO}[NO]} \tag{S14}$$

If we make the assumption that at the inflection point $\frac{k_L[\text{ISOPOOH}]}{k_{RO_2\ +\ HO_2}[HO_2]} \sim \frac{k_L[\text{IHN}]}{k_{RO_2\ +\ NO}[NO]}$ then this expression can be simplified to Equation S15.

$$\frac{\alpha_6 k_{RO_2\ +\ NO} \cdot [NO]}{k_{RO_2\ +\ HO_2} \cdot [HO_2]} = \frac{k_{OH\ +\ IHN} \cdot [\text{IHN}]}{k_{OH\ +\ ISOPOOH} \cdot [\text{ISOPOOH}]} \tag{S15}$$

In Figure S7, at the inflection point [ISOPOOH] = [IHN], therefore, we can simplify this equation further to Equation 2.

G Volatility

Volatility and concentration ultimately dictate a species' ability to condense. Volatility measurements of the second-generation compounds driving new particle formation are lacking in literature, especially at temperatures of the upper troposphere. Therefore, we have calculated volatilities, using SIMPOL [8] with the known functional group contributions at 300 K and applied the Clausius-Clapeyron equation in order to account for temperature using coefficients from Epstein et al. [9]. Using SIMPOL at 300 K, the −OOH group contributes -2.42 to v*b(300), in comparison to $-ONO_2$ and −OH groups which contribute -2.17 and -2.16, respectively. Applied to our system, the dihydroperoxide, $C_5H_{12}O_6$ has a saturation mass concentration, $log\ c^{\circ}(223K)$ = -7.19, compared to the dinitrate, $C_5H_{10}N_2O_8$, $log\ c^{\circ}(223K)$ = -6.33, and the mononitrate $C_5H_{11}NO_7$ falls in between, $log\ c^{\circ}(223K)$ = -6.75, under these volatility calculations, Figure S9. The volatilities of $C_5H_{12}O_6$ and $C_5H_{11}NO_7$, calculated using the Tmax from the FIGAERO CIMS [10], differ to the SIMPOL predictions as seen in Figure S9. $C_5H_{12}O_6$ measured to have a lower volatility ($log\ c^{\circ}(223K)$ = -7.86) in comparison to SIMPOL and $C_5H_{11}NO_7$ to have a higher volatility ($log\ c^{\circ}(223K)$ = -5.91) in comparison, indicating the high uncertainty of calculated SIMPOL values at 300 K extrapolated to 223 K. There were no stages where a volatility for the dinitrate $C_5H_{10}N_2O_8$ could be calculated. This suggests the replacement of an −OOH group with an $-ONO_2$ group increases volatility by roughly two orders of magnitude as expected by the higher hydrogen-bonding capability of an −OOH group. In SIMPOL there is no functionality $-O_2NO_2$, therefore, we have used a combination of R−OO−R (-0.40) and $R-NO_2$ (-2.09) functionality, which together contribute -2.49, lowering the volatility similar to that of an −OOH group. Therefore, under upper-tropospheric conditions, where RO_2NO_2 lifetime to decomposition is longer, condensation of RO_2NO_2 onto particles may become important, rivalling that of non-nitrates. However, it is difficult to experimentally confirm this as the RO_2NO_2 groups are thermally unstable so cannot be verified by FIGAERO [11].

Figure S9 displays the oxidation state as defined in Kroll et al. [12] against the volatility of $C_5H_{12}O_6$, $C_5H_{11}NO_7$ and $C_5H_{10}N_2O_8$. Oxidation state utilises the O:C, H:C and N:C ratio of a sum formula in order to distinguish different levels of oxidation.

$$\text{OSc} = 2 \cdot \text{O:C} - \text{H:C} - 5 \cdot \text{N:C} \tag{S16}$$

Under these definitions, non-nitrates are more oxidised and have lower volatilities than their nitrate counterparts.

It is not suitable to use the elemental parameterisation for volatility from the Volatility Basis Set (VBS) in this instance. VBS is characterised for alpha-pinene and is useful for generalising average functionality. In this isoprene system, the average functionality is different due to the lack of autoxidation at low temperatures and multiple $OH^{\bullet}$ oxidations leading to a greater ratio of −OH groups to −OOH groups laid out in the VBS for α-pinene. In addition, due to the well-studied nature of isoprene chemistry and the smaller subset of molecules, we are able to identify individual functional groups in the compounds. Therefore using generalised averages does not pose any benefits. A correction to the VBS parameterisation would be useful for an isoprene $OH^{\bullet}$ initiated chemistry.

An important measure for a species' contribution to condense onto aerosol is the level of supersaturation. Supersaturation is a function of volatility and concentration and can be represented by the saturation ratio (S^*). The saturation ratio for a compound i is defined as $S_i^* = \frac{[c_i] \cdot m_i}{N_A \cdot C_i^*}$, where $[c_i]$ is the concentration, m_i is the molecular mass and C_i^* is the saturation mass concentration of compound i. In Figure S10, we show the total saturation ratio of $C_5H_{12}O_6$, $C_5H_{11}NO_7$ and $C_5H_{10}N_2O_8$ combined as a function of $HO_2^{\bullet}$:$NO^{\bullet}$. We observe an increasing saturation ratio with increasing $HO_2^{\bullet}$:$NO^{\bullet}$, explaining the better nucleating efficiency of non-nitrates observed in Shen and Russell et al. [3]. However, for all $HO_2^{\bullet}$:$NO^{\bullet}$ the saturation ratio is greater than one, therefore, placing the system in a supersaturated environment. Further study on the volatilities of these three compounds is required to quantify their relative contribution to growth.

H Predicted Flux

The mechanism depicted in Figure 1 starts from isoprene and creates six distinct products through nine pathways. The flux of each pathway can be used to compare the weight and branching of each pathway under the scenarios created for Figure 6 (b-g).

$$\text{Flux}(i) = (k_{IP} \cdot [OH^{\bullet}][IP]) \cdot (k_X[X]) \cdot (k_Y \cdot [OH^{\bullet}]) \cdot (k_Z[Z]) \tag{S17}$$

Where k_{IP}, k_X, k_Y, and k_Z are the reaction rate coefficients for Isoprene + $OH^{\bullet}$, $ISOPOO^{\bullet}$ + $A^{\bullet}$, first generation product (Y) Y + $OH^{\bullet}$ and the second generation $RO_2^{\bullet}$ (Z) $Z^{\bullet}$ + $A^{\bullet}$. A is one of $HO_2^{\bullet}$, $NO^{\bullet}$ and $NO_2^{\bullet}$.

However, as explained above there are uncertainties associated with reaction rate coefficients, k_X and k_Z. Instead relative reaction rate coefficients can be substituted in by dividing the whole equation by $(k_{NO}[NO])^2$ to account for two sets of termination. This transforms Equation S17 into Equation S18, Equation S19, Equation S20, Equation S21 for $C_5H_{10}N_2O_8$, $C_5H_{11}NO_7$(N), $C_5H_{12}O_6$ and $C_5H_{11}NO_8$(P), respectively.

$$\text{Relative Flux}(C_5H_{10}N_2O_8) = (k_{IP} \cdot [OH^{\bullet}][IP]) \cdot (k_{IHN} \cdot [OH^{\bullet}]) = k_{IP}k_{IHN} \cdot [OH^{\bullet}]^2[IP] \tag{S18}$$

$$
\begin{aligned}
\text{Relative Flux}(C_5H_{11}NO_7(N)) &= \frac{(k_{IP}\cdot[OH^{\bullet}][IP])\cdot(k_{IHN}\cdot[OH^{\bullet}])\cdot(k_{HO2}[HO_2^{\bullet}])}{(k_{NO}[NO^{\bullet}])} \\
&= k_{IP}k_{IHN}[OH^{\bullet}]^2[IP]\frac{k_{HO_2}[HO_2^{\bullet}]}{k_{NO}[NO^{\bullet}]} \\
&= k_{IP}k_{IHN}[OH^{\bullet}]^2[IP]\cdot T_{HO_2}\frac{[HO_2^{\bullet}]}{[NO^{\bullet}]}
\end{aligned} \tag{S19}
$$

$$
\begin{aligned}
\text{Relative Flux}(C_5H_{12}O_6) &= \frac{(k_{IP}\cdot[OH^{\bullet}][IP])\cdot(k_{HO_2}[HO_2^{\bullet}])\cdot(k_{ISOPOOH}\cdot[OH^{\bullet}])\cdot(k_{HO2}[HO_2^{\bullet}])}{(k_{NO}[NO^{\bullet}])^2} \\
&= k_{IP}k_{ISOPOOH}[OH^{\bullet}]^2[IP]k_{HO_2}[HO_2^{\bullet}]\frac{(k_{HO_2}[HO_2^{\bullet}])^2}{(k_{NO}[NO^{\bullet}])^2} \\
&= k_{IP}k_{ISOPOOH}[OH^{\bullet}]^2[IP]\cdot T_{HO_2}^2\frac{[HO_2^{\bullet}]^2}{[NO^{\bullet}]^2}
\end{aligned} \tag{S20}
$$

$$
\begin{aligned}
\text{Relative Flux}(C_5H_{11}NO_8(P)) &= \frac{(k_{IP}\cdot[OH^{\bullet}][IP])\cdot(k_{NO_2}[NO_2^{\bullet}])\cdot(k_{IHPN}\cdot[OH^{\bullet}])\cdot(k_{HO2}[HO_2^{\bullet}])}{(k_{NO}[NO^{\bullet}])^2} \\
&= k_{IP}k_{IHPN}[OH^{\bullet}]^2[IP]k_{HO_2}[HO_2^{\bullet}]\frac{(k_{HO_2}k_{NO_2}[HO_2^{\bullet}][NO_2^{\bullet}])}{(k_{NO}[NO^{\bullet}])^2} \\
&= k_{IP}k_{ISOPOOH}[OH^{\bullet}]^2[IP]\cdot T_{HO_2}T_{NO_2}\frac{[HO_2^{\bullet}][NO_2^{\bullet}]}{[NO^{\bullet}]^2}
\end{aligned} \tag{S21}
$$

Here we use known reaction rate coefficients for k_{IP} and $k_{ISOPOOH}$ in combination with reaction rate ratios from Figure 4 and concentrations taken from the global model. Concentrations correspond to the mean output concentrations from the global model at an isothermal surface of 223 K above the Amazon (latitude = [20,-40], longitude = [-81,-30]) for sunrise, midday and sunset. The relative predicted fluxes can be calculated for each term. Normalising by the sum of all fluxes for the midday section allows for a direct comparison in Figure S14.

Table S1: A comparison of reaction rate coefficients of $RO_2^\bullet$ with $NO_2^\bullet$ and $NO^\bullet$. Literature reaction rate coefficients for two carbon fluorinated peroxy radicals with $NO_2^\bullet$ and $NO^\bullet$. ‡Reaction rate coefficient derived in SI sect B. All reaction rate coefficients are in (cm^3s^{-1}).

$RO_2^\bullet$	T (K)	$k_{RO_2 + NO_2}$	$k_{RO_2 + NO}$	$\frac{k_{NO_2}}{k_{NO}}$
**$CF_3CF(CF_3)OO^\bullet$**	296	4.8×10^{-12} [13]	2.1×10^{-11} [13]	0.23
**$CF_3CF_2OO^\bullet$**	298	1.0×10^{-12} [14]	1.1×10^{-11} [15]	0.09
**$CF_3CF_2OO^\bullet$**	225	9.0×10^{-13} [14]	8.3×10^{-12} [15]	0.11
**$CF_3CH_2OO^\bullet$**	296	5.8×10^{-12} [16]	1.2×10^{-11} [16]	0.48
**$CF_3CFClOO^\bullet$**	295	5.9×10^{-12} [17]	1.5×10^{-11} [17]	0.39
**$CF_3C(O)OO^\bullet$**	295	6.6×10^{-12} [18]	2.7×10^{-11} [19]	0.24
**Isoprene $RO_2^\bullet$**	223	3.4×10^{-12}‡	1.3×10^{-11} [1]	0.26

Table S2: Rate coefficients from Wennberg et al.[1]. †There is a lack of literature values describing the rate coefficient of IHPN + $OH^\bullet$, therefore, we are assigning the same rate coefficient as IHN + $OH^\bullet$. ‡For the $RO_2^\bullet$ + $NO_2^\bullet$ rate coefficient a kinetic reasoning has been carried out in SI sect. B.

Reaction	Rate equation	**T = 223K** (cm^3s^{-1})
**ISOPOOH + $OH^\bullet$**	$1.7 \times 10^{-11}\ e^{390/T}$	9.8×10^{-11}
**IHN + $OH^\bullet$**	$8.4 \times 10^{-12}\ e^{390/T}$	4.8×10^{-11}
**IHPN + $OH^\bullet$†**	-	4.8×10^{-11}
**IEPOX + $OH^\bullet$**	$5.82 \times 10^{-11}\ e^{-400/T}$	9.7×10^{-12}
**$RO_2^\bullet$ + $HO_2^\bullet$**	$2.1 \times 10^{-13}\ e^{1300/T}$	7.2×10^{-11}
**$RO_2^\bullet$ + $NO^\bullet$**	$2.7 \times 10^{-12}\ e^{350/T}$	1.3×10^{-11}
**$RO_2^\bullet$ + $NO_2^\bullet$‡**	-	3.4×10^{-12}

Table S3: Fits from Figure 2 (a). Fits in the form of the equation C_5 IP-OOM fraction = a log[$OH^\bullet$] + b

Group	a	b
Saturated	0.32	-1.81
Unsaturated	-0.23	2.00
Other	-0.09	0.81

Table S4: Isomers from kinetic model used in Figure 5. Names used in the reaction mechanism described above for $C_5H_{12}O_6$, $C_5H_{11}NO_7$ and $C_5H_{10}N_2O_8$.

Sum formula	Isomers	Mechanism names
**$C_5H_{12}O_6$**	3	'ISOP1OH2OOH3OH4OOH', 'ISOP1OH2OOH3OOH4OH', 'ISOP1OOH2OH3OOH4OH'
**$C_5H_{11}NO_7$**	10	'ISOP1N2OH3OH4OOH', 'ISOP1N2OH3OOH4OH', 'ISOP1N2OOH3OH4OH','ISOP1OH2N3OH4OOH', 'ISOP1OH2N3OOH4OH', 'ISOP1OH2OH3OOH4N', 'ISOP1OH2OOH3N4OH', 'ISOP1OH2OOH3OH4N', 'ISOP1OOH2OH3N4OH','ISOP1OOH2OH3OH4N'
**$C_5H_{10}N_2O_8$**	6	'ISOP1N2N3OH4OH', 'ISOP1N2OH3N4OH', 'ISOP1N2OH3OH4N', 'ISOP1OH2N3N4OH', 'ISOP1OH2N3OH4N', 'ISOP1OH2OH3N4N'

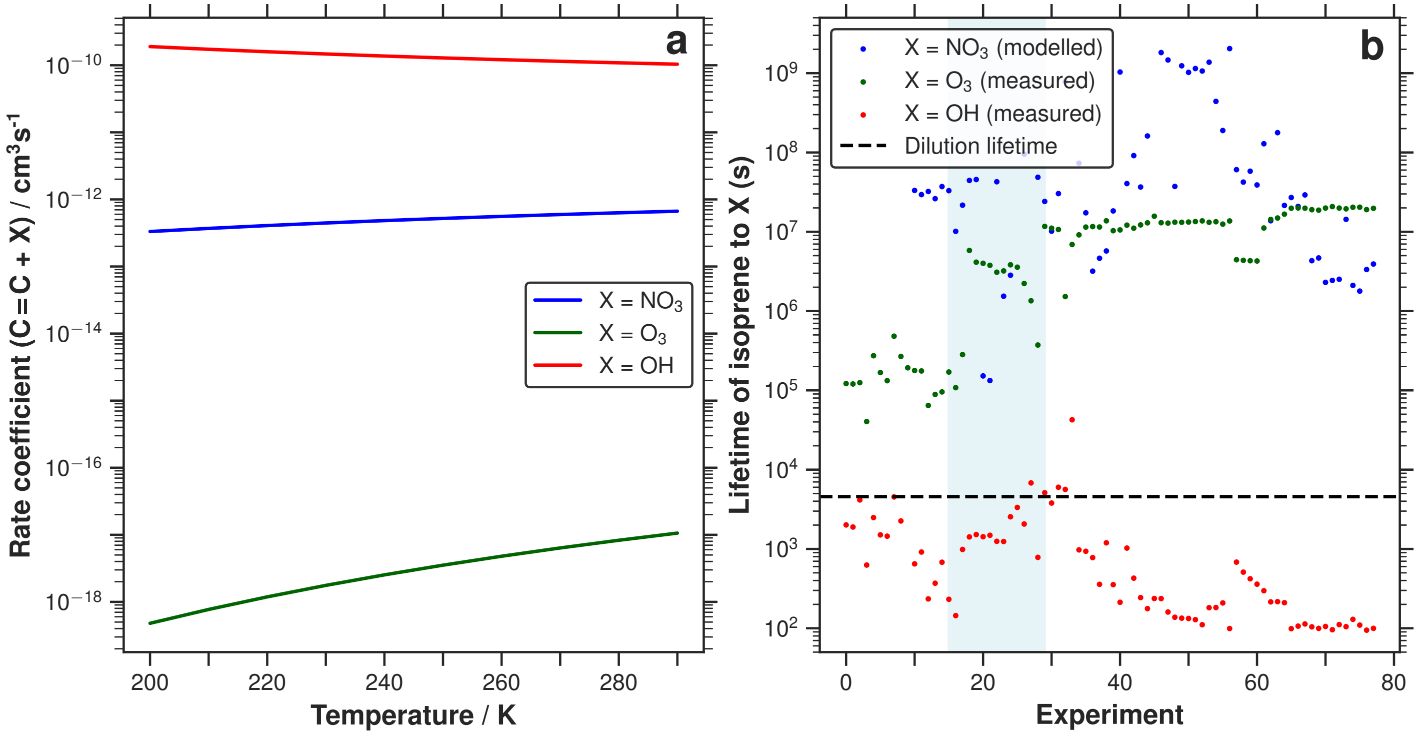

Figure S1: Kinetic comparison of isoprene oxidation: (a) Isoprene reaction rate coefficients with oxidative radicals (O_3, $NO_3^{\bullet}$, and $OH^{\bullet}$) versus temperature. The reaction rate coefficients are from the International Union of Pure and Applied Chemistry (IUPAC) Task Group on Atmospheric Chemical Kinetic Data Evaluation with equations $k_{O_3} = 1.05\times10^{-14} \cdot e^{\frac{-2000}{T}}$ cm^3s^{-1}, $k_{NO_3} = 2.95\times10^{-12} \cdot e^{\frac{-450}{T}}$ cm^3s^{-1} and $k_{OH} = 2.7\times10^{-11} \cdot e^{\frac{390}{T}}$ cm^3s^{-1}. (b) The lifetime of isoprene to oxidative radicals are plotted for each steady-state stage in CLOUD 16. Lifetime of isoprene to X = $\frac{1}{k_X[X]}$. For $OH^{\bullet}$ and O_3, concentrations were measured (see Methods for details). However, there was no $NO_3^{\bullet}$ measurement; in place, a kinetic model using measured $NO^{\bullet}$, $NO_2^{\bullet}$, $OH^{\bullet}$, $HO_2^{\bullet}$, O_3, H_2O, isoprene was run to determine the approximate $NO_3^{\bullet}$ concentration, further details are included in Methods. Most data is at 223 K but the blue shaded area highlights experiments at 243 K. The horizontal black dashed line indicates the largest physical loss of isoprene in the chamber, the dilution lifetime of the CLOUD chamber, at $\sim$ 76 minutes.

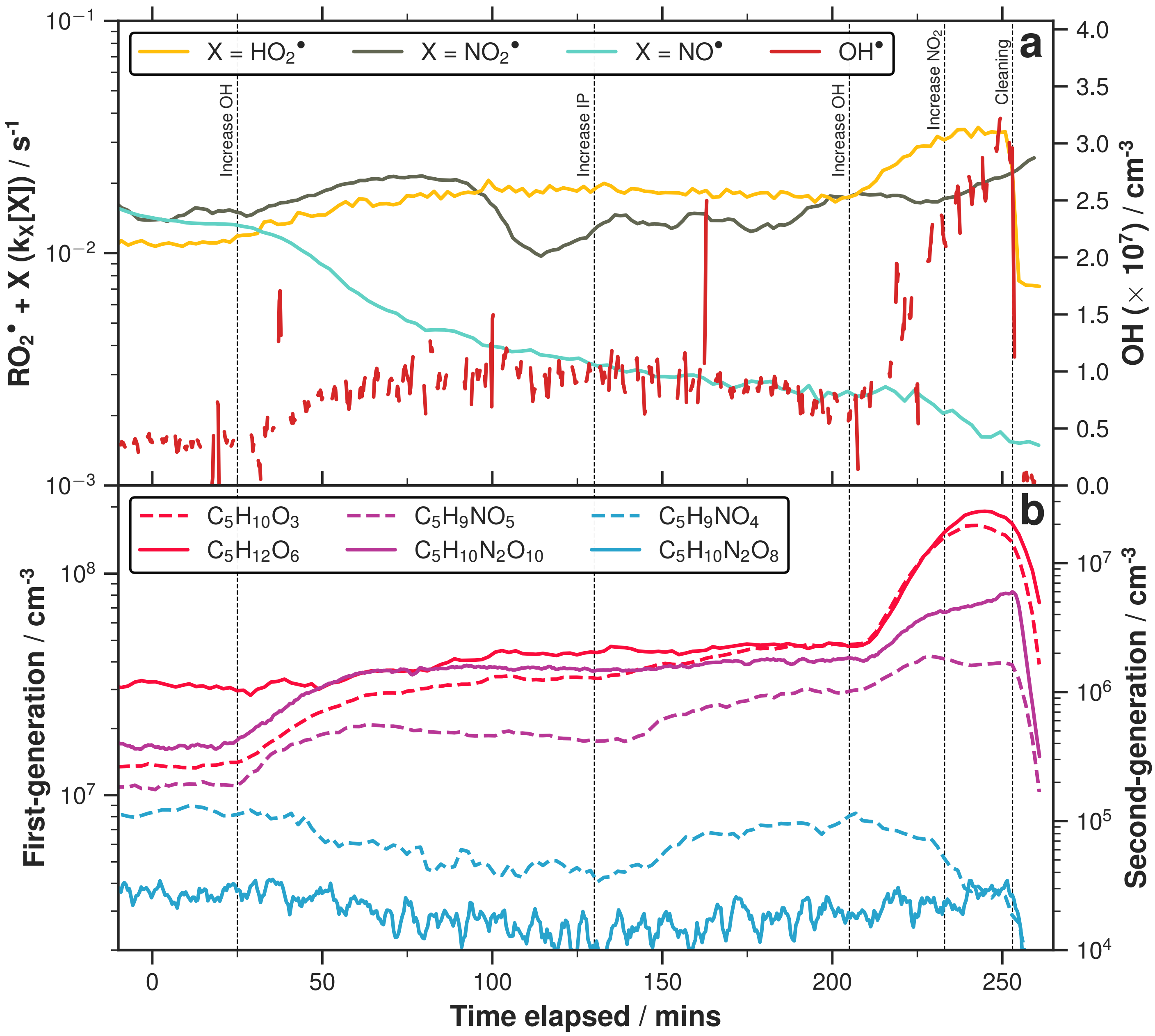

Figure S2: Peroxynitrate (RO_2NO_2) formation. An isoprene + $OH^\bullet$ oxidation experiment with $HO_2^\bullet$, $NO_2^\bullet$ and low $NO^\bullet$. (a) displays the reactivity ($k_X[X]$) of $HO_2^\bullet$ (yellow), $NO_2^\bullet$ (grey) and $NO^\bullet$ (blue) towards $RO_2^\bullet$ on the left axis. Additionally on the right axis, the concentration of $OH^\bullet$ (red) is used to indicate the approximate concentration of $RO_2^\bullet$ present. (b), first (dashed, left axis) and second-generation (right axis) isoprene oxidation product time series. Described in Figure 1, compounds shown are $C_5H_{10}O_3$ and $C_5H_{12}O_6$ (red) for $HO_2^\bullet$ termination, $C_5H_9NO_4$ and $C_5H_{10}N_2O_8$ (blue) for $NO^\bullet$ termination, and $C_5H_9NO_5$ and $C_5H_{10}N_2O_{10}$ (purple) for $NO_2^\bullet$ termination. In the absence of $NO^\bullet$, the dinitrate ($C_5H_{10}N_2O_8$) appears at background level whereas the peroxynitrate ($C_5H_{10}N_2O_{10}$) is on a similar order of magnitude to dihydroperoxide ($C_5H_{12}O_6$). [$C_5H_{10}O_3$, $C_5H_9NO_4$, $C_5H_9NO_5$ and $C_5H_{12}O_6$] are from the Br-MION2-CIMS and [$C_5H_{10}N_2O_8$ and $C_5H_{10}N_2O_{10}$] are from the NO_3-CIMS. Vertical black dashed lines indicate changes in experimental conditions. Here, "Cleaning" refers to the low-pressure mercury lamp being turned off and chamber fans set to 100%.

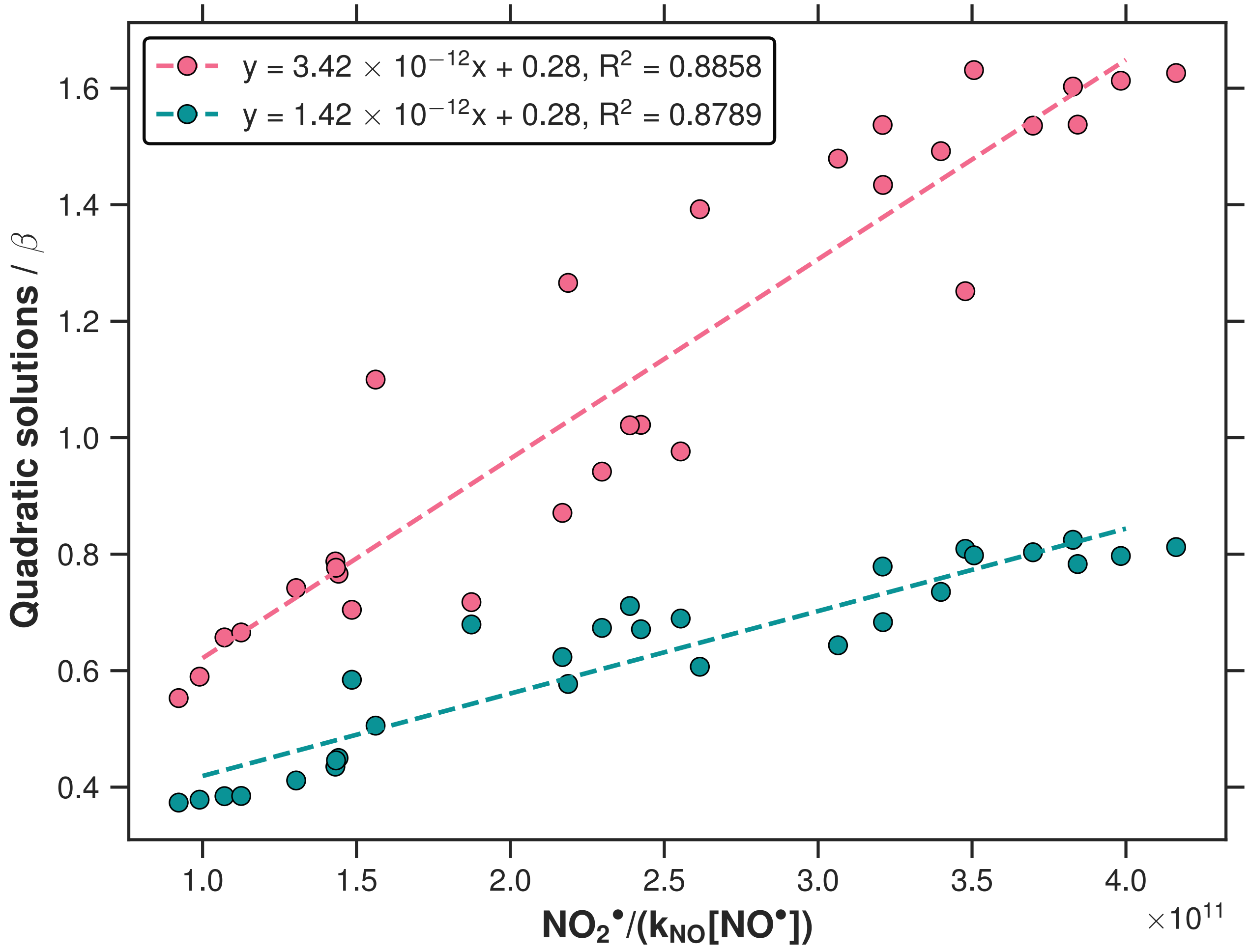

Figure S3: Rate coefficient of peroxynitrate formation. Quadratic solutions, β from solving the Equation S7 as a function of $\frac{[NO_2^\bullet]}{k_{NO}[NO^\bullet]}$. The equation was solved for each steady-state chemistry stage, and the two roots were plotted in pink and blue. Comparing the calculated β with the measured $\frac{[NO_2^\bullet]}{k_{NO}[NO^\bullet]}$ should give k_{NO_2} as the gradient. Two fits through the data have been plotted with equations: $\beta = 3.42 \times 10^{-12} \frac{[NO_2^\bullet]}{k_{NO}[NO^\bullet]} + 0.28$ (pink) and $\beta = 1.42 \times 10^{-12} \frac{[NO_2^\bullet]}{k_{NO}[NO^\bullet]} + 0.28$ (blue).

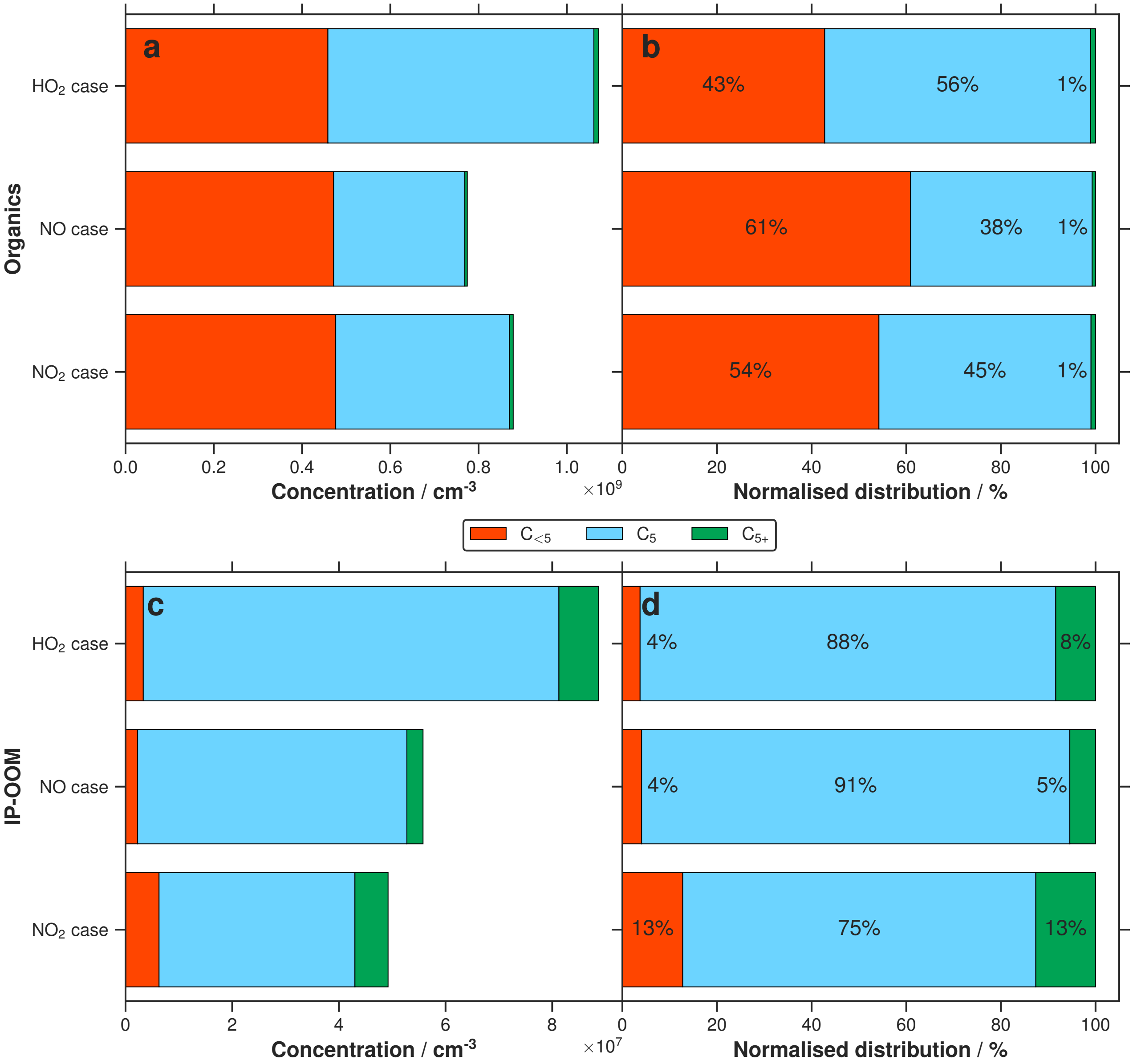

Figure S4: Organic distribution between fragments ($n_C < 5$), monomers (n_C = 5) and dimers ($n_C > 5$) for each case. (a) and (b) highlight the total concentration and percentage contribution to the total signal of each group, respectively - fragments (red), monomers (blue) and dimers (green) - for all organic species. (c) and (d) describe the total concentration and percentage contribution of each group to the total signal for IP-OOM (isoprene oxygenated organic molecules, $n_C > 3$, $n_O^{eff} > 3$). The total concentration for the HO_2, NO and NO_2 cases, respectively, are $1.1{\times}10^9$, $7.7{\times}10^8$, $8.8{\times}10^8$ cm^{-3} for all organics; $8.9{\times}10^7$, $5.6{\times}10^7$ and $4.9{\times}10^7$ cm^{-3} for IP-OOM. Therefore, the IP-OOM percentage of total organics are 8.3, 7.2 and 5.6% for the HO_2, NO and NO_2 cases.

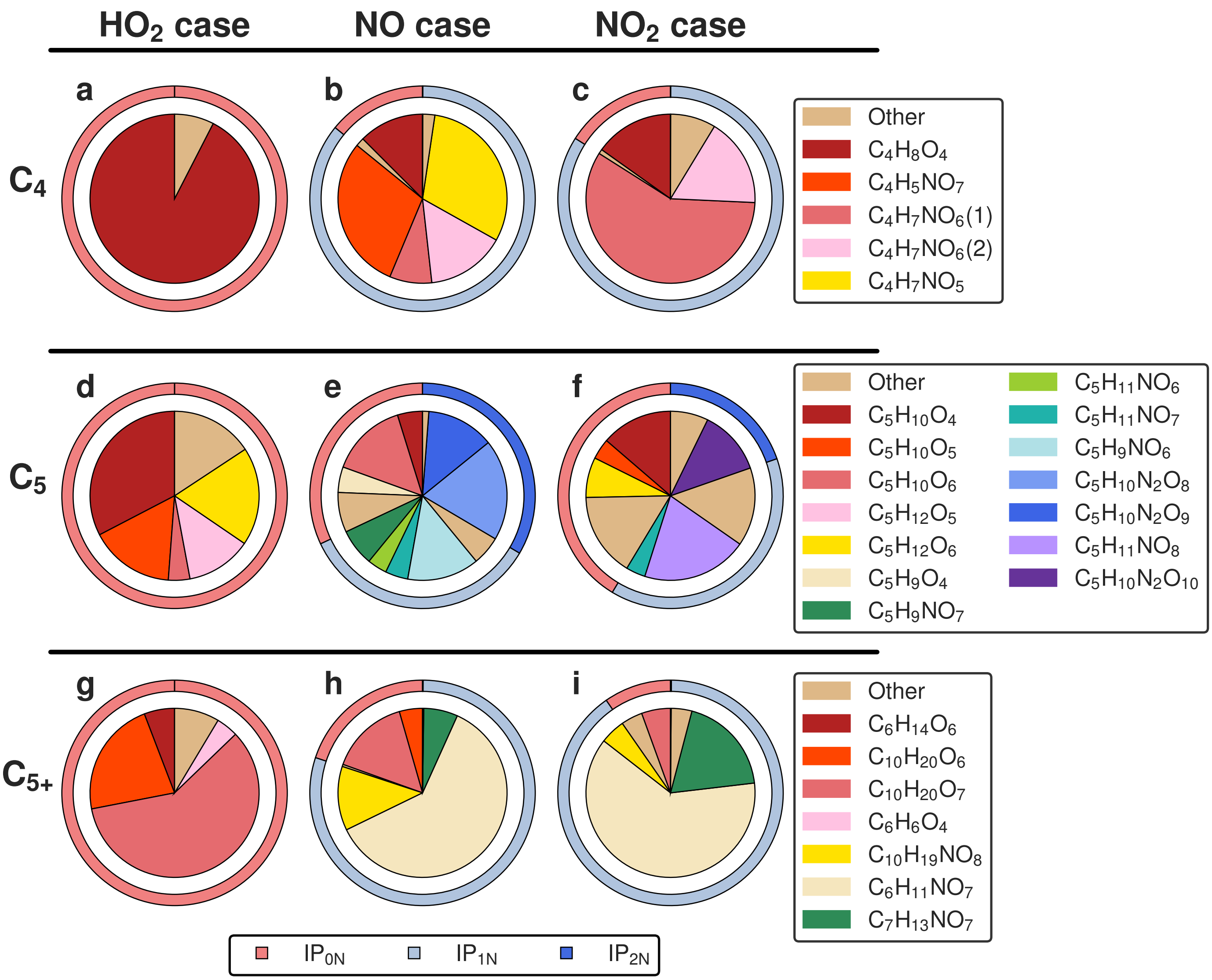

Figure S5: Case study IP-OOM distribution. (a), (b) and (c) are speciated distribution of C_4 fragments; (d), (e) and (f) for C_5 monomers; (g), (h) and (i) for C_{5+} dimers, for the HO_2, NO and NO_2 cases, respectively. The outer circle demonstrates the proportion of IP_{0N} (red, n_N = 0), IP_{1N} (light blue, n_N = 1), and IP_{2N} (dark blue, n_N = 2) classes that make up that IP-OOM (isoprene oxygenated organic molecules, $n_C > 3$, $n_O^{eff} > 3$) section. The area of each segment corresponds to the percentage contribution to that IP-OOM section. The inner circle gives the molecular breakdown of IP_{0N}, IP_{1N} and IP_{2N} and aligns with the outer circle. Compounds below 3.5 % of the signal are grouped and placed in “Other” for that class. The colours of segments within rows correspond to the same compounds described in the legend. This is a combination of three mass spectrometers applying NO_3^-, Br^- and NH_4^+ ionisation methods.

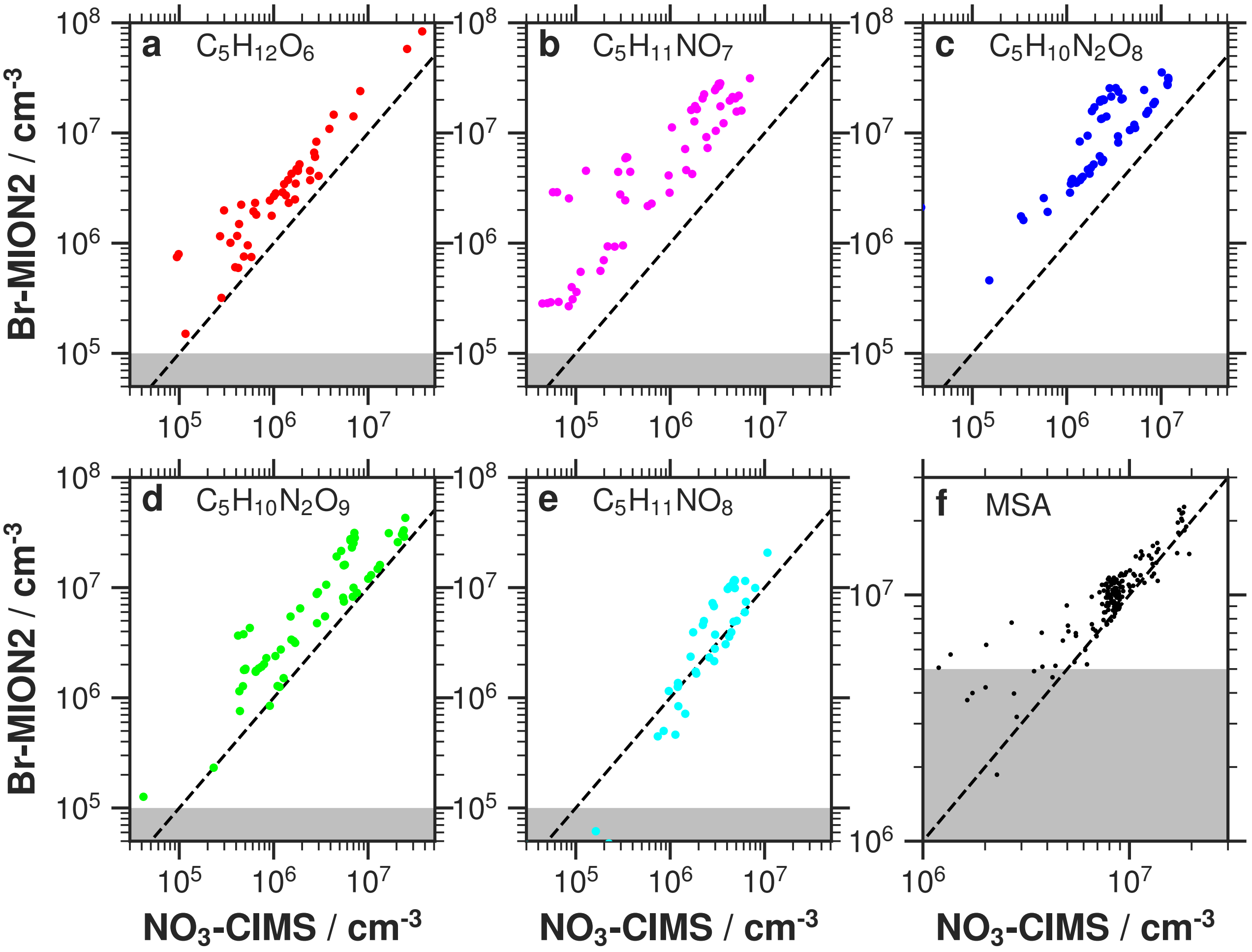

Figure S6: Sensitivity comparison between the Br-MION2-CIMS and the NO_3-CIMS. (a), (b), (c), (d), (e) and (f) plots concentration of Br^- detection versus NO_3^- for $C_5H_{12}O_6$, $C_5H_{11}NO_7$, $C_5H_{10}N_2O_8$, $C_5H_{10}N_2O_9$, $C_5H_{11}NO_8$ and CH_4SO_3. The dashed line represents the 1:1 line and the grey shaded area indicates the limit of detection of the Br-MION2-CIMS, the limit of detection for NO_3-CIMS is below 5×10^4 cm^{-3}. (a-e) are averaged steady-state chemistry stages. (f) is the second time resolution for methane sulfonic acid (MSA) at 223 K within the chamber. For MSA, the Br-MION2-CIMS experienced a larger limit of detection around 5×10^6 cm^{-3}.

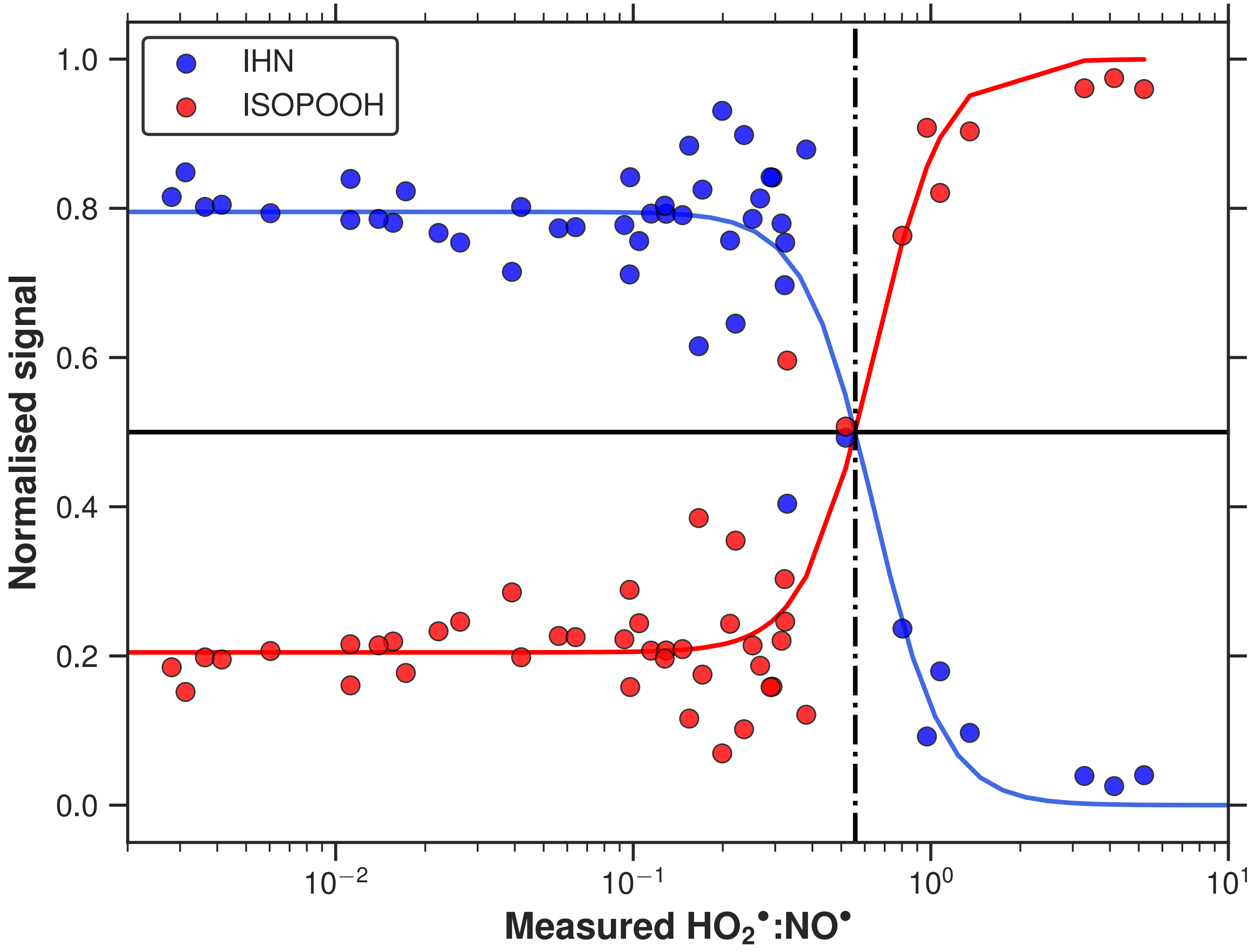

Figure S7: ISOPOOH and IHN ratio as a function of radical ratio. The fraction of ISOPOOH (isoprene hydroxy hydroperoxide, $C_5H_{10}O_3$, see SI sect. A for details) and IHN (isoprene hydroxy nitrate, $C_5H_9NO_4$) to each other: $[ISOPOOH]_{norm} = \frac{[ISOPOOH]}{[ISOPOOH]+[IHN]}$, $[IHN]_{norm} = \frac{[IHN]}{[ISOPOOH]+[IHN]}$ plotted against the $HO_2^{\bullet}$:$NO^{\bullet}$. Data at 223 K are plotted and fits through the data are added. The data splits into two different regimes: $NO^{\bullet}$-dominated and $HO_2^{\bullet}$-dominated. At a certain point a critical threshold is reached where the ratio drops steeply as switches regimes, from IHN to ISOPOOH dominated regime. A horizontal black line is added when $[ISOPOOH]_{norm} = [IHN]_{norm} = 0.5$ and a vertical dashed-dotted line indicates this point on the x-axis. This critical threshold occurs at $\frac{HO_2^{\bullet}}{NO^{\bullet}} = 0.56$.

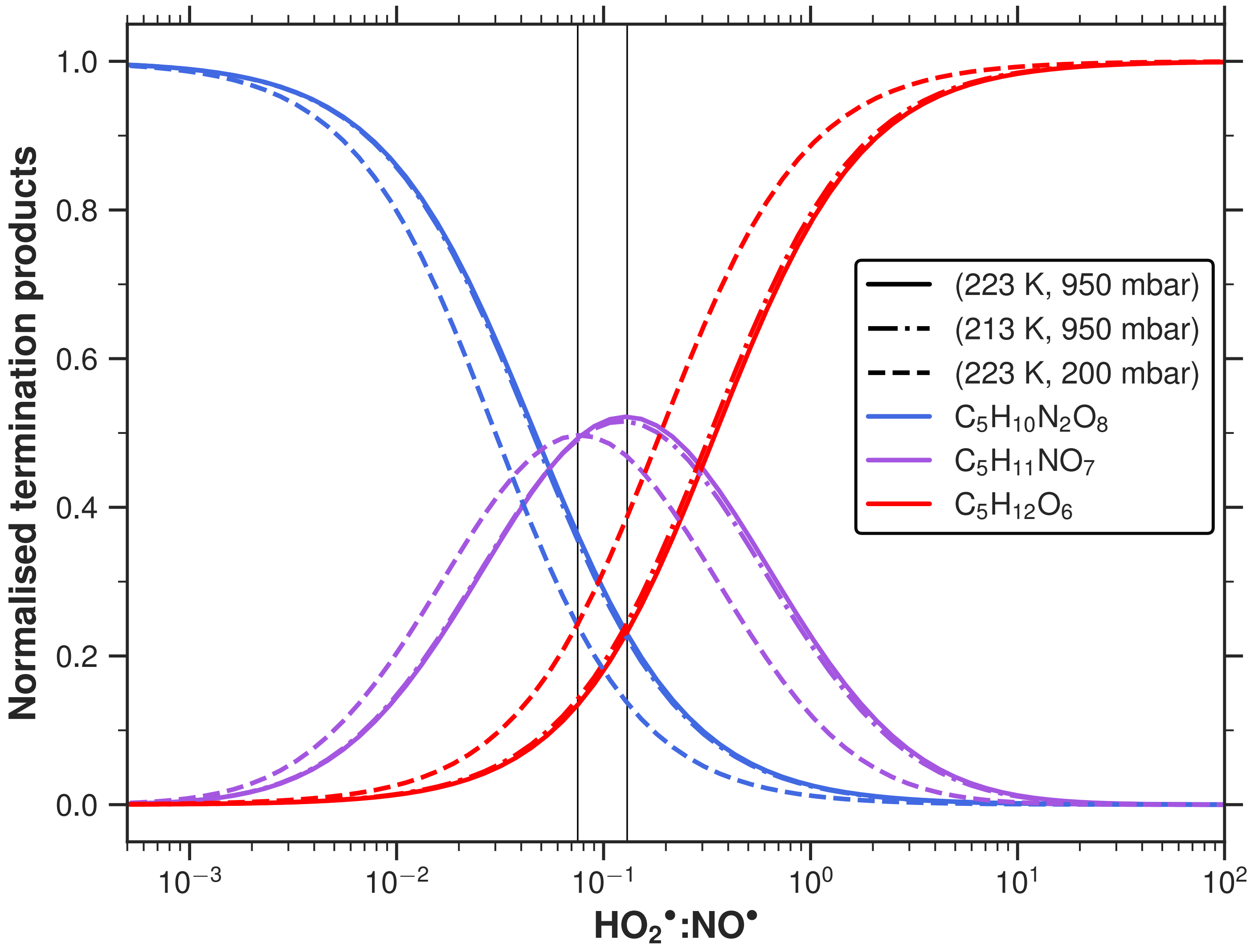

Figure S8: Pressure and temperature effect on the second-generation products in the isoprene mechanism. A kinetic model using the AMORE mechanism (see Methods for details) was run with three different temperature and pressure settings in order to determine the difference between CLOUD (223 K and 950 mbar) and the aircraft (213 K and 187 mbar) [20] for $C_5H_{10}N_2O_8$ (blue), $C_5H_{11}NO_7$ (purple), and $C_5H_{12}O_6$ (red). (1) 223 K and 950 mbar (solid); (2) 213 K and 950 mbar (dashed-dotted); (3) 223 K and 200 mbar (dashed). The effect of 10 K decrease in temperature is negligible for the product distribution. However, a 750 mbar pressure change moves the point of equal termination (represented by vertical black lines) from 0.13 to 0.075, roughly a factor of two. The rest of the curves follow a similar trend. Therefore, in order to adjust qualitatively for the pressure difference, the aircraft data could be shifted to the right by a factor of two in $HO_2^\bullet$:$NO^\bullet$.

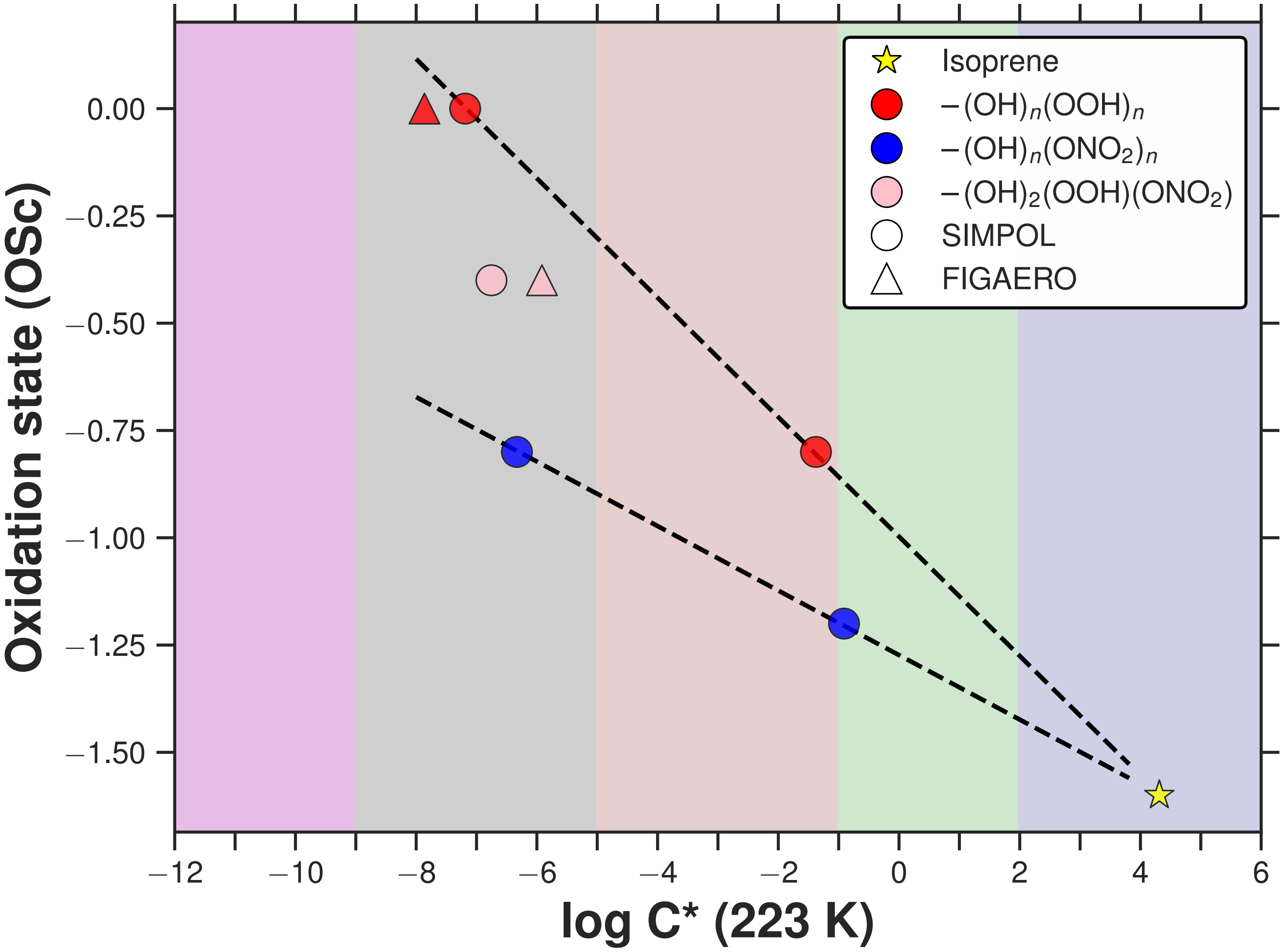

Figure S9: Volatility estimates of $HO_2^{\bullet}$ and $NO^{\bullet}$ termination products. The 2D VBS indicates the difference in oxidation state and volatility of nitrate groups and hydroperoxide groups on the isoprene backbone. Volatilities (circles, star) are calculated through SIMPOL at 300 K [8] and extrapolated to 223 K via the Clausius-Clapeyron equation. The oxidation state follows the definition from Kroll et al. [12] detailed in SI sect. G. The star (yellow) represents the oxidation state and volatility of isoprene, C_5H_8. The two red circles represent the first and second-generation oxidation products, ISOPOOH (isoprene hydroxy hydroperoxide, $C_5H_{10}O_3$) and $C_5H_{12}O_6$, indicating the change in this phase space by an $OH^{\bullet}$ oxidation followed by a $HO_2^{\bullet}$ termination. The two blue circles represent IHN (isoprene hydroxy nitrate, $C_5H_9NO_4$) and $C_5H_{10}N_2O_8$, the analogous pathway with an $OH^{\bullet}$ oxidation step and a $NO^{\bullet}$ termination instead. The pink circle represents the cross-product mononitrate ($C_5H_{11}NO_7$). The dashed lines drawn to guide the eye as to the difference between the two oxidation pathways and the coloured background indicates the volatility class (ULVOC, ELVOC, LVOC, SVOC, IVOC). Additionally, volatilities calculated from the T_{max} in the FIGAERO (triangles) have been added for $C_5H_{12}O_6$ and $C_5H_{11}NO_7$.

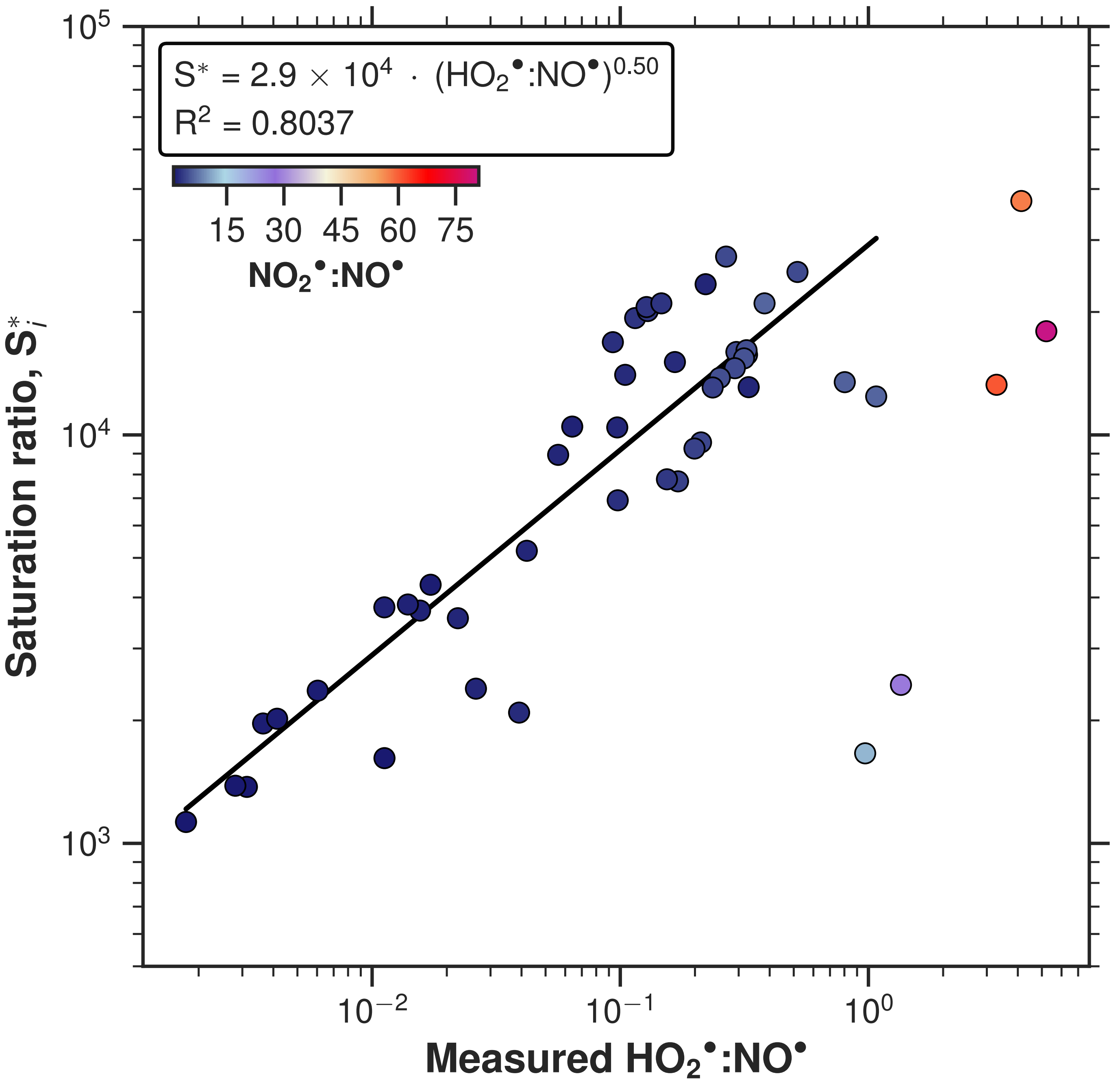

Figure S10: Total saturation ratio of $C_5H_{12}O_6$, $C_5H_{11}NO_7$, and $C_5H_{10}N_2O_8$ as a function of $HO_2^\bullet:NO^\bullet$ at 223 K. Total saturation ratio, S^* (as defined in SI sect. G) of $C_5H_{12}O_6$, $C_5H_{11}NO_7$ and $C_5H_{10}N_2O_8$ combined as a function of $HO_2^\bullet:NO^\bullet$. The colour represents the $NO_2^\bullet:NO^\bullet$ ratio and the black line is a fit through data with $NO_2^\bullet:NO^\bullet < 10$. Volatilities are calculated using SIMPOL [8] at 300 K and converting to 223 K with the Clausius-Clapeyron equation (see SI sect. G for more details).

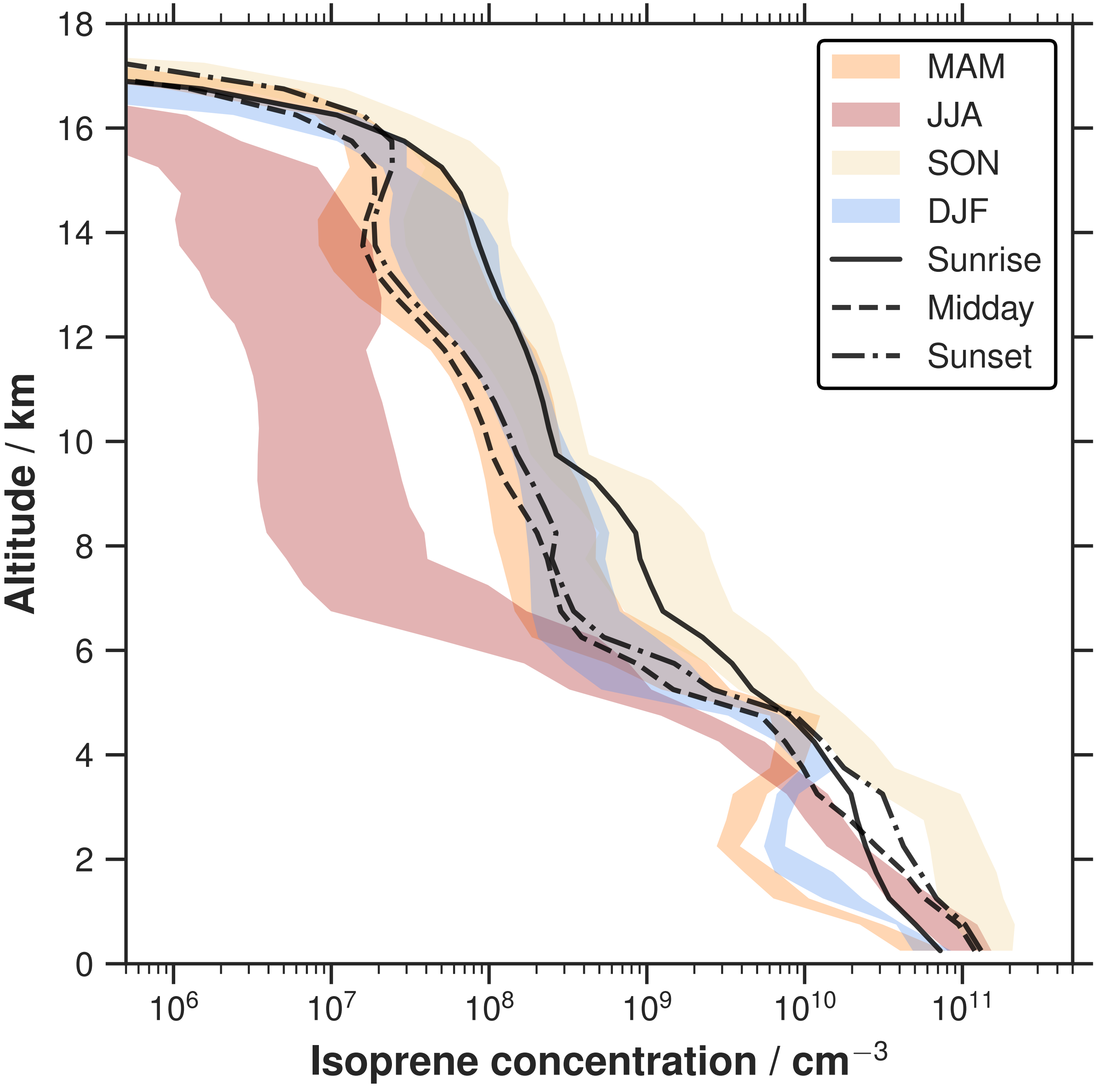

Figure S11: Isoprene vertical profile above the ATTO tower. An annually averaged isoprene vertical profile (black) of the column above the ATTO tower (latitude = [-1.865,-3.731], longitude = [-57.1875, -59.0625]) for the three time periods sunrise (solid), midday (dashed), and sunset (dot-dashed). Additionally, the shaded areas represent the seasonally averaged isoprene vertical profiles: MAM (orange), JJA (red), SON (beige), DJF (blue). These are shaded between minimal and maximal values for sunrise, midday and sunset.

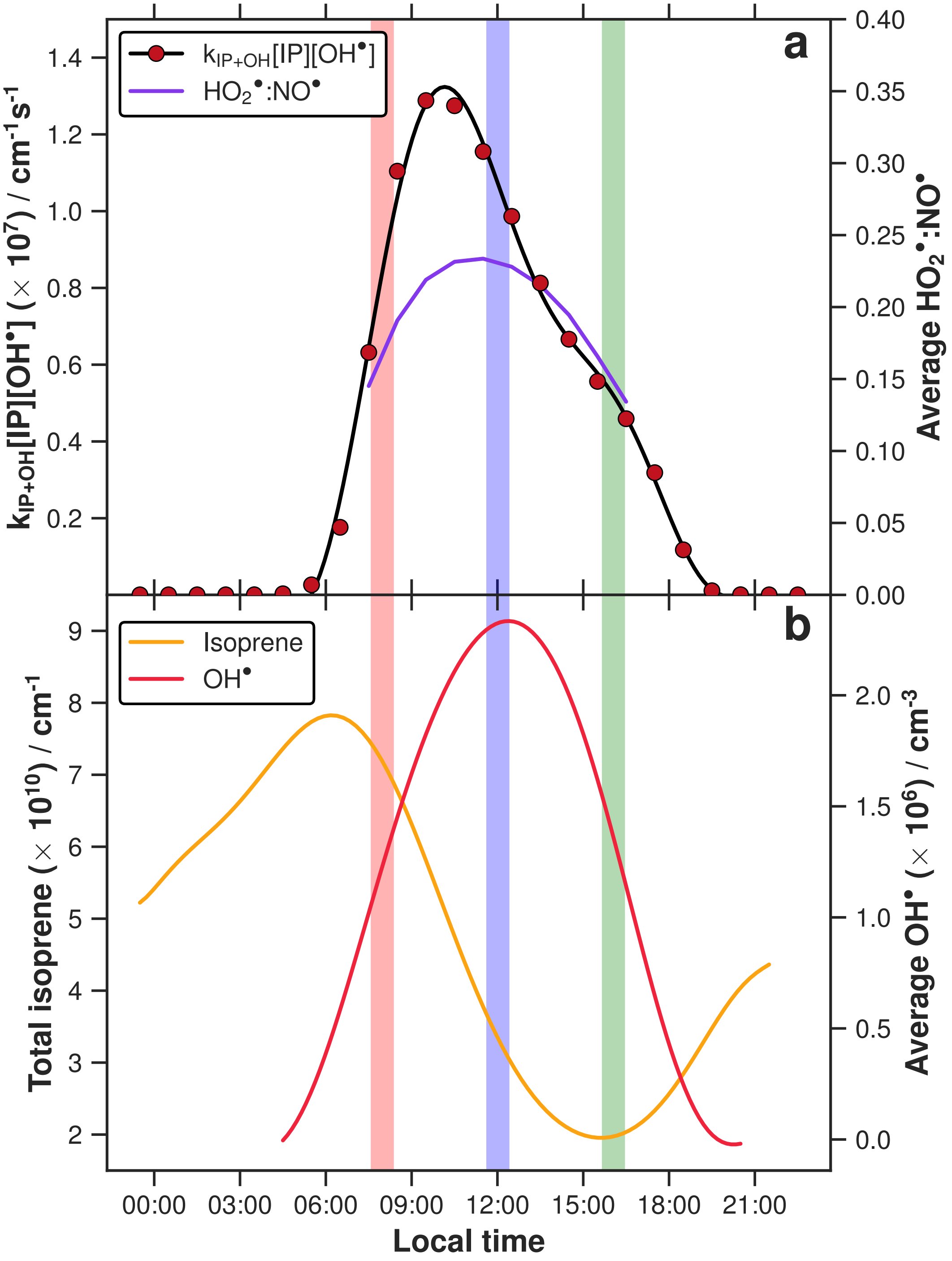

Figure S12: Total isoprene oxidation rate for the upper troposphere above the Amazon. (a) Annually averaged diurnal cycle of isoprene oxidation rate (black) summed over the Amazon rainforest using a global model. Total isoprene oxidation rate (first-generation production rate) is defined as the product of isoprene concentration, hydroxyl radical concentration and isoprene + OH• reaction rate coefficient (k_{IP+OH}) summed between longitudes (-81,-30) and latitudes (-40,20) at a 223 K isothermal surface ranging between 200-300 mbar, the region plotted in Figure 6 (b-g). Additionally, the average ratio of HO_2• concentration to NO• concentration (purple) for this area is plotted. (b), shows the total concentration for isoprene (yellow) and average OH• concentration (pink) over this area for the same time period. Each line represents a fit through hourly data points (shown for first-generation production rate) of an annual average iso-thermal surface at the temperature of 223 K for the Amazon region. Highlighted regions red (sunrise), blue (midday) and green (sunset) represent the hour time periods used in Figure 6 (b-g). Details on the simulation can be found in Methods.

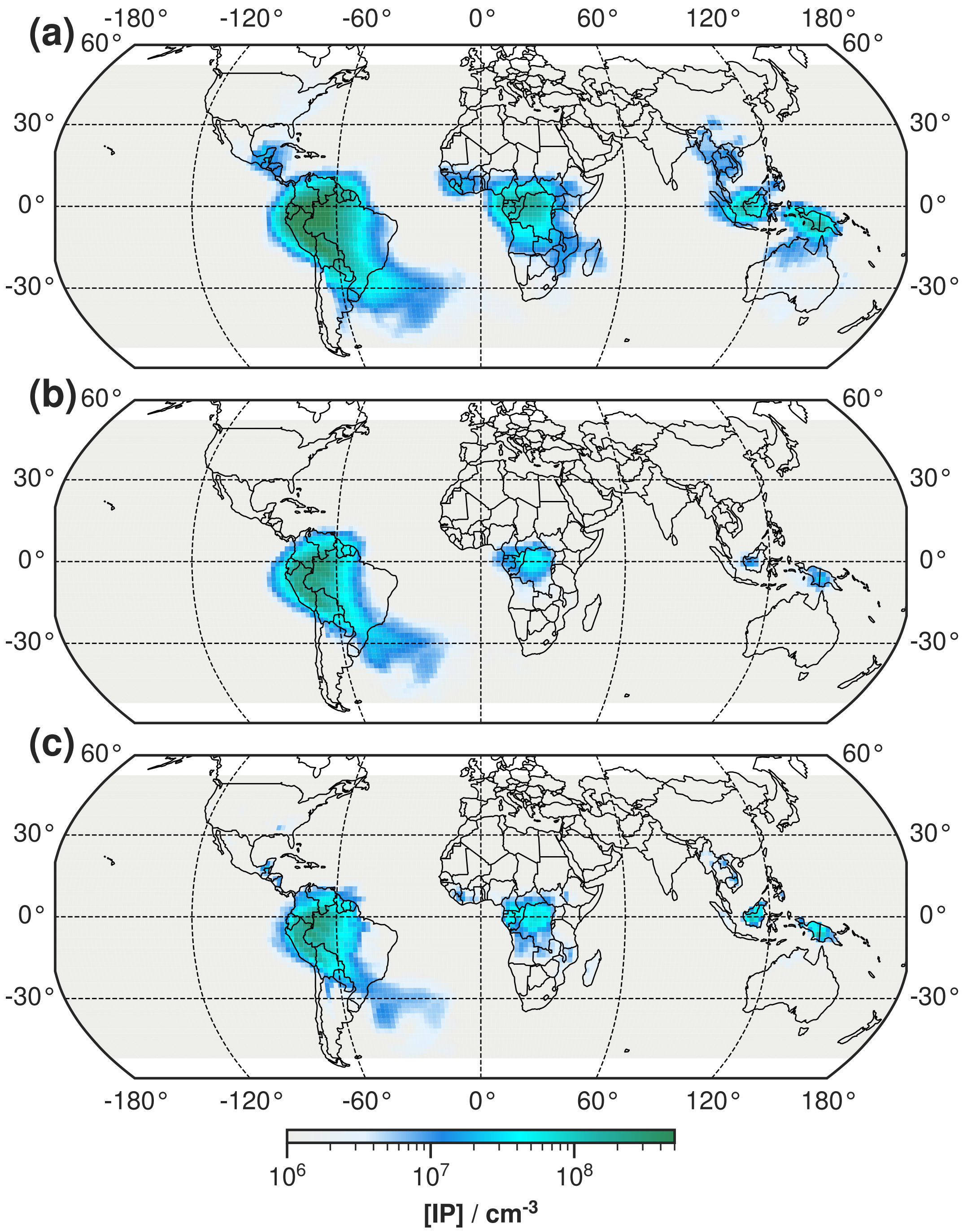

Figure S13: Global isoprene isothermal-surface distribution at 223 K. Annually averaged global model (EMAC) of isoprene concentrations in cm^{-3} for (a) sunrise, (b) midday and (c) sunset. Midday is defined using the hour of highest shortwave flux at the top of the atmosphere whereas sunrise and sunset are defined when the shortwave flux is closest to a quarter of the maximum. Using an annual average removes any seasonality bias, however, may reduce peak concentrations in comparison to observational campaigns. Three isoprene hotspots correspond to the three largest tropical rainforests (Amazon, Congo, Papua New Guinea). (a) sunrise exhibits the largest concentrations of isoprene due to nocturnal accumulation because of low rates of oxidation overnight. All data are annually averaged isothermal-surface plots at the temperature of 223 K from the global model (EMAC) ranging between 200 - 300 hPa and (-51.3°, 51.3°) latitude. Details on the simulation can be found in Methods. The map in the figures were made with Natural Earth.

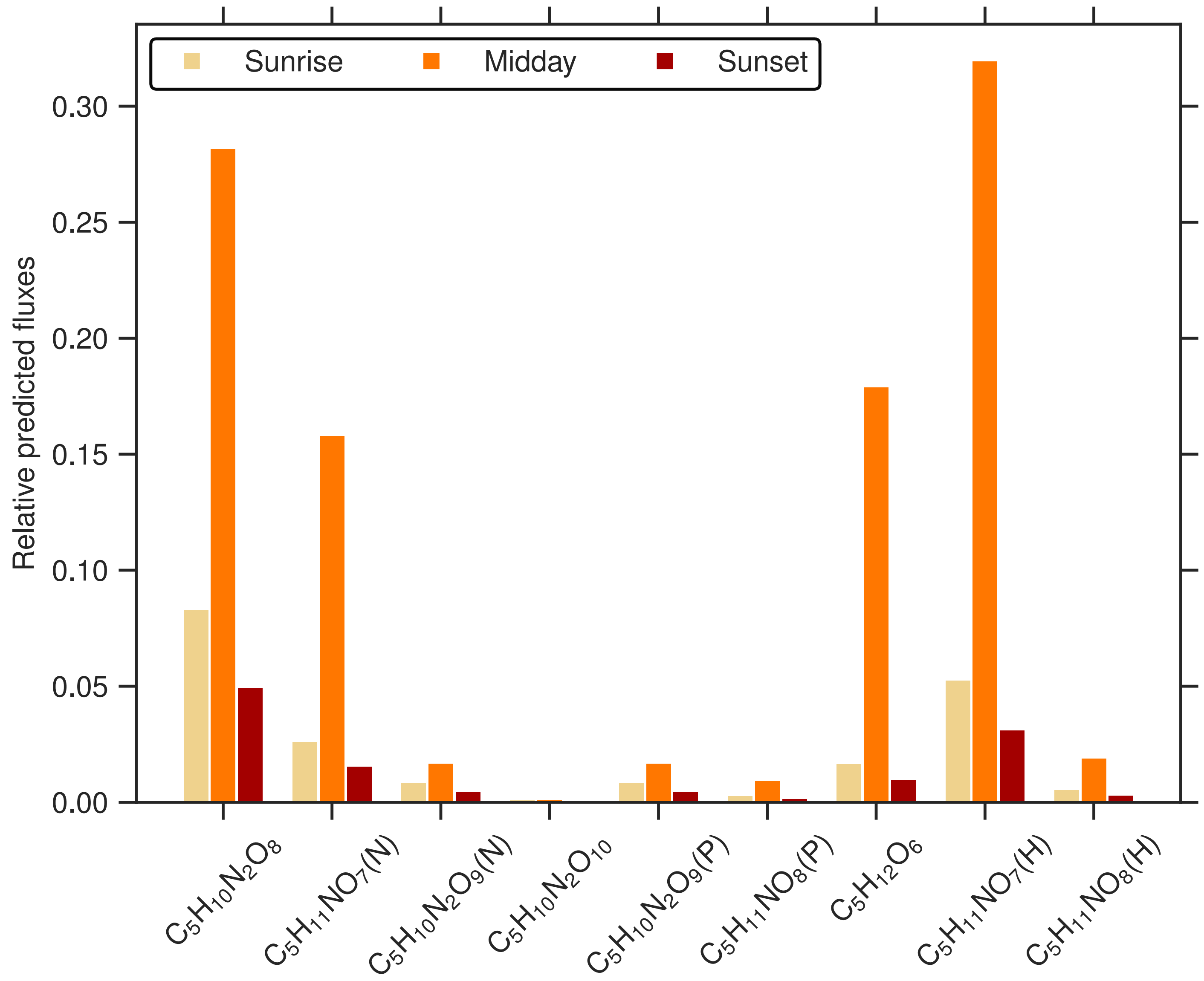

Figure S14: Relative predicted production flux using relative reaction rate coefficients derived from Figure 4. Relative predicted flux, defined in SI sect. H, describes the predicted formation of each second generation species for sunrise (beige), midday (orange), and sunset (red) using the relative reaction rate coefficients derived from Figure 4. The nine species represent the nine different formation mechanisms depicted in Figure 1. For cross products ($C_5H_{11}NO_7$, $C_5H_{11}NO_8$ and $C_5H_{10}N_2O_9$) the first generation intermediate is put in brackets: (N) is IHN (isoprene hydroxy nitrate, $C_5H_9NO_4$), (P) is IHPN (isoprene hydroxy peroxy nitrate, $C_5H_9NO_5$) and (H) is ISOPOOH (isoprene hydroxy hydroperoxide, $C_5H_{10}O_3$). Each bar is normalised to the total midday flux to give an indication of the total organic formation and relative branching.

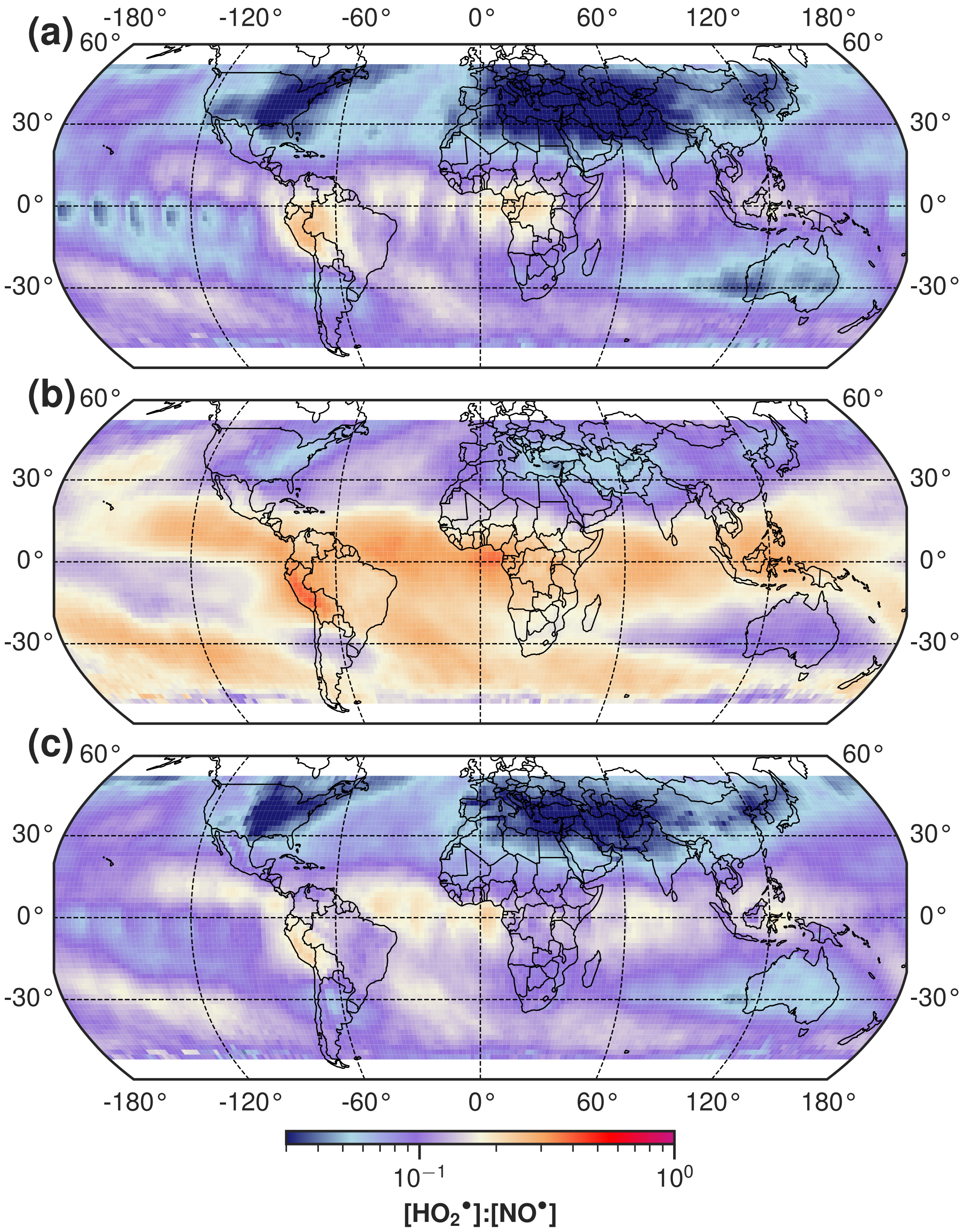

Figure S15: Global isothermal-surface distribution of $HO_2^{\bullet}$:$NO^{\bullet}$ at 223 K from EMAC global model. Annually averaged global distributions of $HO_2^{\bullet}$:$NO^{\bullet}$ for (a) sunrise, (b) midday and (c) sunset. Midday is defined using the hour of highest shortwave flux at the top of the atmosphere, sunrise and sunset are defined when the shortwave flux is closest to a quarter of the maximum. Time periods were determined for each hour time zone, therefore the wavelike structure observed over the tropical ocean arises from rapid changes in this ratio at sunrise and sunset within the region defined in the same time zone, this is more evident over the oceans due to smaller values of both HO_x and NO_x. All data are annually averaged isothermal-surface plots at the temperature of 223 K from the global model (EMAC) ranging between 200 - 300 hPa and (-51.3°, 51.3°) latitude. Details on the simulation can be found in Methods. The map in the figures were made with Natural Earth.

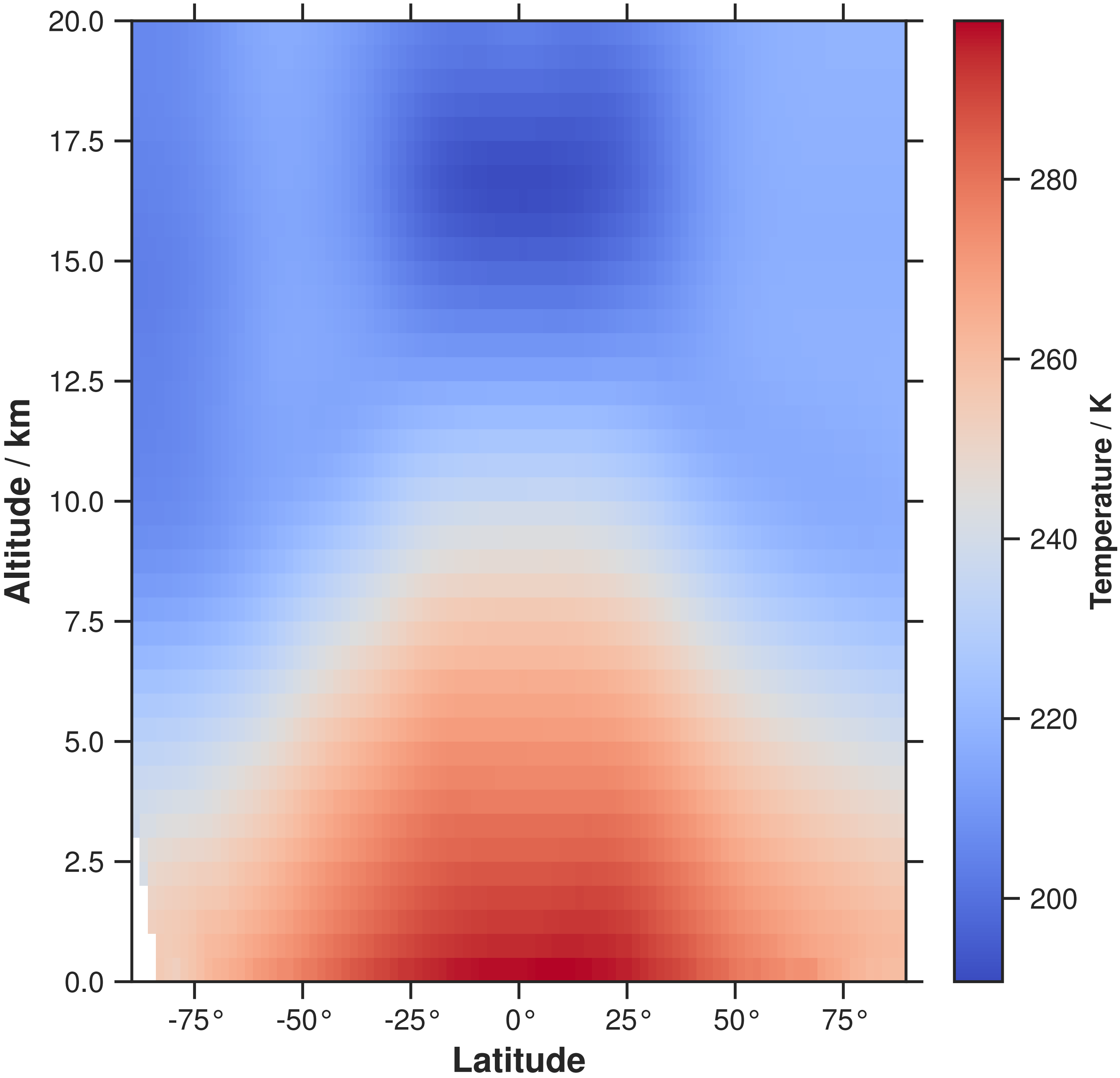

Figure S16: Standard temperature profile. The annually averaged global model output temperature as a function of latitude and altitude. Further details on the simulation can be found in the Methods section.

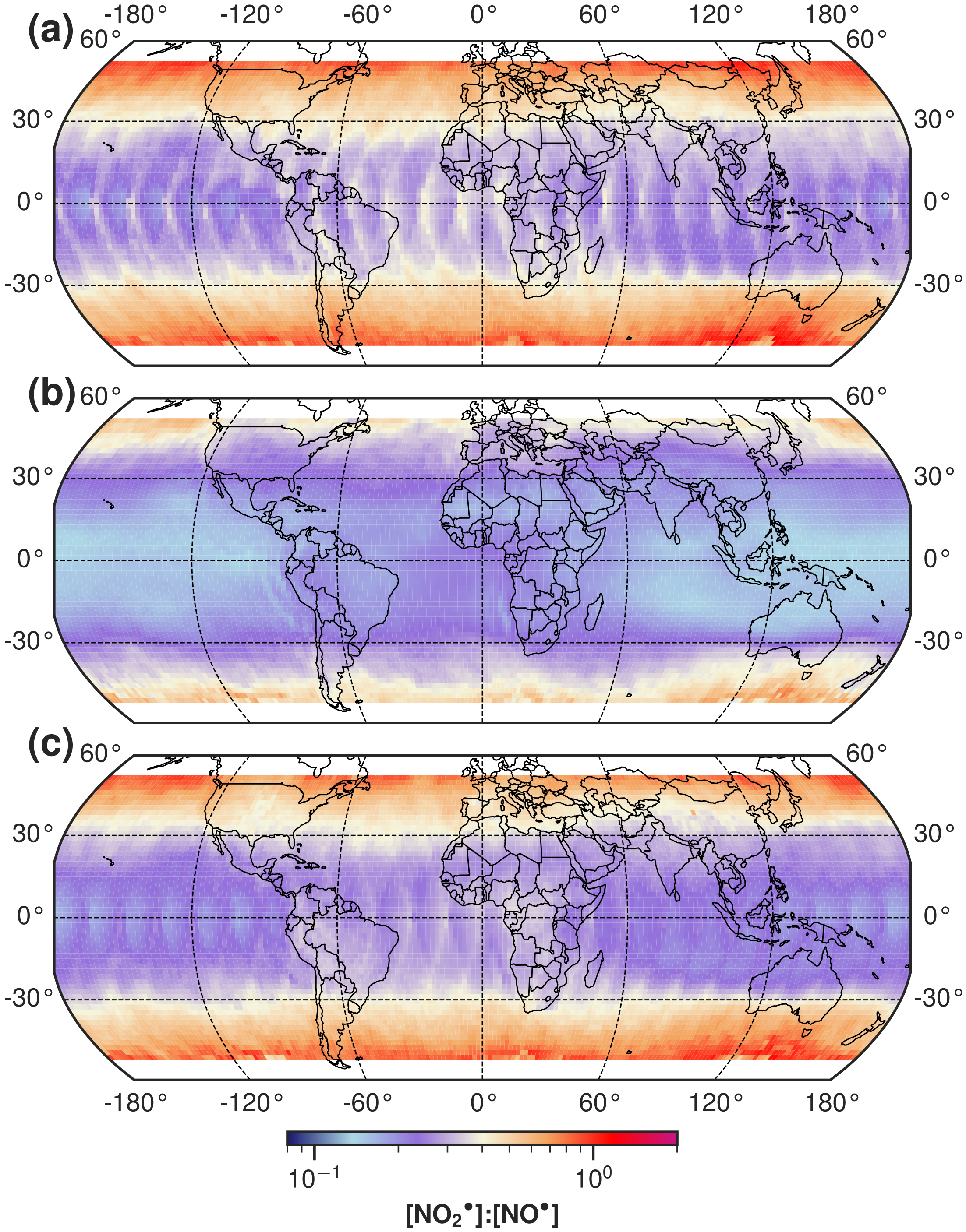

Figure S17: Global isothermal-surface distribution of $NO_2^{\bullet}$:$NO^{\bullet}$ at 223 K from EMAC global model. Annually averaged global distributions of $NO_2^{\bullet}$:$NO^{\bullet}$ for (a) sunrise, (b) midday and (c) sunset. Midday is defined using the hour of highest shortwave flux at the top of the atmosphere, sunrise and sunset are defined when the shortwave flux is closest to a quarter of the maximum. Time periods were determined for each hour time zone. All data are annually averaged isothermal-surface plots at the temperature of 223 K from the global model (EMAC) ranging between 200 - 300 hPa and (-51.3°, 51.3°) latitude. Details on the simulation can be found in Methods. The map in the figures were made with Natural Earth.

Supplementary References

1. Wennberg, P. O. *et al.* Gas-Phase Reactions of Isoprene and Its Major Oxidation Products. *Chemical Reviews* **118,** 3337–3390. ISSN: 0009-2665 (Apr. 2018).
2. He, X.-C. *et al.* Characterisation of gaseous iodine species detection using the multi-scheme chemical ionisation inlet 2 with bromide and nitrate chemical ionisation methods. English. *Atmospheric Measurement Techniques* **16,** 4461–4487. ISSN: 1867-1381 (Oct. 2023).
3. Shen, J. *et al.* New particle formation from isoprene under upper-tropospheric conditions. en. *Nature* **636,** 115–123. ISSN: 1476-4687 (Dec. 2024).
4. Fischer, E. V. *et al.* Atmospheric peroxyacetyl nitrate (PAN): a global budget and source attribution. English. *Atmospheric Chemistry and Physics* **14,** 2679–2698. ISSN: 1680-7316 (Mar. 2014).
5. Tyndall, G. S. *et al.* Atmospheric chemistry of small organic peroxy radicals. *Journal of Geophysical Research: Atmospheres* **106,** 12157–12182 (2001).
6. Kirkby, J. *et al.* Role of sulphuric acid, ammonia and galactic cosmic rays in atmospheric aerosol nucleation. en. *Nature* **476,** 429–433. ISSN: 1476-4687 (Aug. 2011).
7. D'Ambro, E. L. *et al.* Molecular composition and volatility of isoprene photochemical oxidation secondary organic aerosol under low- and high-NO_x conditions. English. *Atmospheric Chemistry and Physics* **17,** 159–174. ISSN: 1680-7316 (Jan. 2017).
8. Pankow, J. F. & Asher, W. E. SIMPOL.1: a simple group contribution method for predicting vapor pressures and enthalpies of vaporization of multifunctional organic compounds. English. *Atmospheric Chemistry and Physics* **8,** 2773–2796. ISSN: 1680-7316 (May 2008).
9. Epstein, S. A., Riipinen, I. & Donahue, N. M. A Semiempirical Correlation between Enthalpy of Vaporization and Saturation Concentration for Organic Aerosol. *Environmental Science & Technology* **44,** 743–748. ISSN: 0013-936X (Jan. 2010).
10. Bannan, T. J. *et al.* A method for extracting calibrated volatility information from the FIGAERO-HR-ToF-CIMS and its experimental application. English. *Atmospheric Measurement Techniques* **12,** 1429–1439. ISSN: 1867-1381 (Mar. 2019).
11. Lee, B. H. *et al.* Highly functionalized organic nitrates in the southeast United States: Contribution to secondary organic aerosol and reactive nitrogen budgets. EN. *Proceedings of the National Academy of Sciences* **113,** 1516–1521 (Feb. 2016).
12. Kroll, J. H. *et al.* Carbon oxidation state as a metric for describing the chemistry of atmospheric organic aerosol. en. *Nature Chemistry* **3,** 133–139. ISSN: 1755-4349 (Feb. 2011).
13. Møgelberg, T. E., Sehested, J., Bilde, M., Wallington, T. J. & Nielsen, O. J. Atmospheric Chemistry of CF3CFHCF3 (HFC-227ea): Spectrokinetic Investigation of the CF3CFO2•CF3 Radical, Its Reactions with NO and NO2, and Fate of the CF3CFO•CF3 Radical. *The Journal of Physical Chemistry* **100,** 8882–8889. ISSN: 0022-3654 (Jan. 1996).
14. Badenes, M. P. Formation, thermal decomposition and atmospheric implications of the CF2(OH)CF2OONO2 and CF3CF2OONO2 peroxynitrates. A theoretical study. *Chemical Physics Letters* **673,** 68–73. ISSN: 0009-2614. (2025) (Apr. 2017).
15. V. Olkhov, R. & M. Smith, I. W. Time-resolved experiments on the atmospheric oxidation of C 2 H 6 and some C 2 hydrofluorocarbons. en. *Physical Chemistry Chemical Physics* **5,** 3436–3442 (2003).
16. Nielsen, O. J., Gamborg, E., Sehested, J., Wallington, T. J. & Hurley, M. D. Atmospheric Chemistry of HFC-143a: Spectrokinetic Investigation of the CF3CH2O2. bul. Radical, Its Reactions with NO and NO2, and the Fate of CF3CH2O. *The Journal of Physical Chemistry* **98,** 9518–9525 (1994).
17. Sehested, J. UV absorption spectrum of CF3CFClO2 and kinetics of the self reaction of CF3CFCl and CF3CFClO2 and the reactions of CF3CFClO2 with NO and NO2. en. *International Journal of Chemical Kinetics* **26,** 1023–1039. ISSN: 1097-4601 (1994).
18. Wallington, T. J., Sehested, J. & Nielsen, O. J. Atmospheric chemistry of CF3C(O)O2 radicals. Kinetics of their reaction with NO2 and kinetics of the thermal decomposition of the product CF3C(O)O2NO2. *Chemical Physics Letters* **226,** 563–569. ISSN: 0009-2614 (Aug. 1994).
19. Maricq, M. M., Szente, J. J., Khitrov, G. A. & Francisco, J. S. The CF3C(O)O2 Radical. Its UV Spectrum, Self-Reaction Kinetics, and Reaction with NO. *The Journal of Physical Chemistry* **100,** 4514–4520. ISSN: 0022-3654 (Jan. 1996).

20. Curtius, J. *et al.* Isoprene nitrates drive new particle formation in Amazon's upper troposphere. en. *Nature* **636,** 124–130. ISSN: 1476-4687 (Dec. 2024).
